# Flagella-related gene mutations in *Vibrio cholerae* during extended cultivation in nutrient-limited media impair cell motility and prolong culturability

**DOI:** 10.1128/msystems.00109-23

**Published:** 2023-08-29

**Authors:** Kazuhisa Okada, Amonrattana Roobthaisong, Shigeyuki Hamada

**Affiliations:** 1 Research Institute for Microbial Diseases, Osaka University, Suita, Osaka, Japan; 2 Thailand-Japan Research Collaboration Center on Emerging and Re-emerging Infections, National Institute of Health, Nonthaburi, Thailand; University of Wisconsin-Madison, Madison, Wisconsin, USA

**Keywords:** cell motility, flagella-related genes, adaptive mutations, *Vibrio cholerae*, VNC

## Abstract

Understanding the survival strategies of pandemic cholera pathogens in aquatic environments is important for preventing their dissemination. Here, we report a phenomenon wherein long-term cultivation of *Vibrio cholerae* under nutrient-limited condition (M9 minimal medium supplemented with 0.2% glucose) causes mutations in flagella-related genes, thus inhibiting motility. Moreover, the motility defect reduced the transition of the viable but nonculturable state of the organisms. Maintenance of proliferative capability allows for the rapid population growth and prolonged survival in environments with nutrient sources. Diverse flagella-related genes, including *flrA, flrC, flrD, flhA, flhF, fliD, fliF, fliG, fliH, fliI, fliP, fliQ, flgF, flgL, flgK, motX,* and *pomA,* underwent mutations during the cultivation. However, only one gene (site) was mutated in a single *V. cholerae*. Longer cultivation for up to 300 days yielded further mutations in metabolism-related genes and the loss of virulence factors (e.g., CTX phage) and large DNA regions (~35 kb). These mutations were detected in most organisms of *V. cholerae*, accompanying the loss of genomic integrity. Motility-defective variants with mutations in the acetate kinase gene had become predominant in culturable cells after long-term cultivation in independently replicated experiments. These findings shed light on a survival mechanism in which *V. cholerae* adapts to an environmental niche by accumulating mutations.

**IMPORTANCE:**

*Vibrio cholerae* undergoes a transition to a viable but non-culturable (VNC) state when subjected to various environmental stresses. We showed here that flagellar motility was involved in the development of the VNC state of *V. cholerae*. In this study, motility-defective isolates with mutations in various flagella-related genes, but not motile isolates, were predominantly obtained under the stress of long-term batch culture. Other genomic regions were highly conserved, suggesting that the mutations were selective. During the stationary phase of long-term culture, *V. cholerae* isolates with mutations in the acetate kinase and flagella-related genes were predominant. This study suggests that genes involved in specific functions in *V. cholerae* undergo mutations under certain environmental conditions.

## INTRODUCTION

*Vibrio cholerae* is a curved, motile, Gram-negative bacillus that causes cholera, a disease characterized by severe diarrhea in humans (1). Human infections are estimated to be caused by the ingestion of food and water contaminated with *V. cholerae*, killing 95,000 people each year in countries where cholera is endemic. *V. cholerae* serogroup O1 has been classified into two biotypes: classical and El Tor. The classical biotype is thought to be responsible for the first six cholera pandemics since the early 19th century, while the ongoing seventh cholera pandemic, which began in 1961, is caused by the El Tor biotype (2). Genome analysis of the seventh pandemic isolates revealed that the pandemic spread from the Bay of Bengal in at least three independent but overlapping waves with a common ancestor in the 1950s and identified multiple transcontinental transmission events (3). Currently, the classical biotype of *V. cholerae* O1 has disappeared globally (4, 5).

*V. cholerae* has been isolated from aquatic environmental sources where it persists between disease outbreaks (6, 7). *V. cholerae* can enter a viable but non-culturable (VNC) state in response to adverse conditions, such as physiological and abiotic stresses and nutritional restriction (6). In the VNC state, bacteria cannot be cultured on standard growth media but can retain their cellular integrity with reduced metabolic activities, including ATP synthesis. Some VNC cells can be resuscitated back to culturable cells under suitable stimuli (7 – 10). The recent assessment of the cholera epidemic in Haiti, caused by a single-source introduction of *V. cholerae* O1, showed emerging novel lineages in the environment, characterized by mutations in genes potentially involved in adaptive responses (11). Reports indicate that bacteria, including *V. cholerae*, have a global response system that significantly alters gene expression and cellular metabolism to adapt to environmental stress, with their survival accompanying the increase in genetic variations (12, 13).

*V. cholerae* is known to survive in aquatic environments where the supply of nutrients is limited. Thus, it is critically important to elucidate how *V. cholerae* responds to starvation stress under such environments. In this study, we used the M9 medium containing defined inorganic salts and glucose, as a sole carbon source, for the long-term cultivation of the organisms. We also investigated genomic and phenotypic changes, including the VNC state over time, and found that the organisms lost cellular motility accompanying mutations in flagella-related genes that are involved in the prolonged survival of the organisms in the given environment.

## RESULTS AND DISCUSSION

### Decreased culturability/motility during prolonged culture

**Fig 1:**
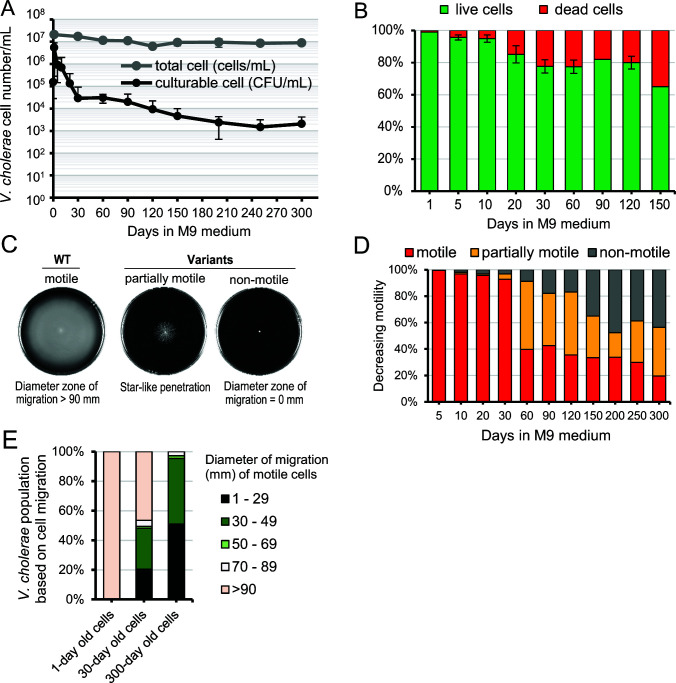
Two-step reduction in culturable cell numbers of *V. cholerae* and changes toward decreased or stopped motility during long-term incubation. (**A**) Culturable cells were counted by plating on LB agar during prolonged incubation in M9 minimal medium, supplemented with 0.2% glucose, at 37°C. Total cell number (both live and dead) was measured using a bacterial counting chamber. The number of culturable cells rapidly decreased from day 1 to day 30, followed by a gradual reduction. Data are expressed as the mean ± standard deviation of five independent experiments. (**B**) Percentage of live and dead cells, determined using the LIVE/DEAD BacLight kit, is shown. Bars represent the mean ± standard deviation of three independent experiments. (**C**) Individual colonies after prolonged culture were inoculated into motility agar plates and incubated at 37°C for 24 hours. Motile phenotype displays the formation of swimming halos that expand from the center of the plate outward. Partially motile variants show a star-like penetration pattern. (**D**) Percentages of motile, partially motile, and non-motile cells were determined using at least 100 randomly selected colonies at the indicated time points. Data show cumulative results from 19 independent experiments. (**E**) Distribution of halo diameter of cell migration at day 1, 30, or 300 was measured, and the percent population of each diameter of migration range is shown. Cumulative data are shown from three independent experiments.

Effects of long-term cultivation of *V. cholerae* in M9 minimal medium (M9) containing 0.2% glucose were observed for up to 300 days. *V. cholerae* was initially inoculated at approximately 10^5^ CFU/mL and incubated at 37°C without shaking. The number of culturable cells increased up to 10^7^ CFU/mL on day 1 of incubation, and thereafter, two distinct phases were noted in the growth curve, a rapid decrease in the initial phase (until day 30) followed by a slow decline. The number of culturable cells decreased and remained at 3 × 10^4^ CFU/mL on day 30 (Fig. 1A) and at 2 × 10^3^ CFU/mL on day 300. The cell viability, determined by staining with SYTO 9 and propidium iodide, decreased to nearly 80% on day 30 and then declined moderately (Fig. 1B). As the proportion of culturable bacteria decreased to approximately 1/200 in the initial phase, it is considered that most living cells transitioned to the VNC state.

**Fig 2:**
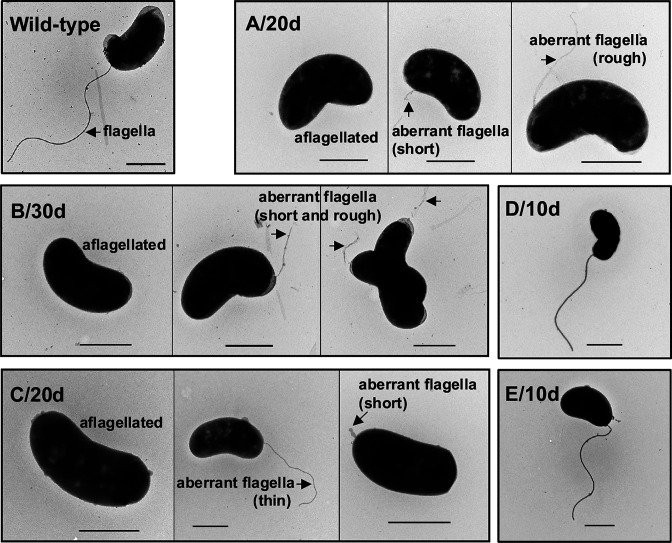
Naturally occurring motility-defective isolates exhibiting aberrant or normal flagella. Transmission electron micrographs of wild-type *V. cholerae* MS84A and five naturally occurring non-motile isolates (A/20d, B/30d, C/20d, D/10d, and E/10d) grown overnight in LB broth. Negative staining was performed using a 2% uranyl acetate solution. Scale bars = 1.0 µM.

When motility of *V. cholerae* in culture was monitored over time, three major bacterial phenotypes were observed on motility agar plates: motile, partially motile (star-like penetration), and non-motile cells (Fig. 1C). On day 5 of culture, 0.1% were partially motile, and 0.4% were non-motile; however, a drastic change in the motile phenotype occurred between days 30 and 60 (Fig. 1D). The motile population also showed a tendency toward shorter movement trajectories over time (Fig. 1E). We tested whether motility could be restored in isolates that developed motility impairment between days 5 and 30 (Fig. S1; Table S1). A total of 11 out of 19 partially motile and 9 out of 37 non-motile isolates reverted to the motile phenotype, whereas 36 isolates did not. Five non-motile isolates were observed under a transmission electron microscope (Fig. 2). WT, D/10d, and E/10d strains showed a single flagellum at the cell pole. In contrast, in A/20d, B/30d, and C/20d, flagella were absent in most cells, and when present, an abnormal polar flagellar structure thinner and shorter than that of the WT strain was observed. Whole-genome sequencing (WGS) of these five isolates revealed that D/10d and E/10d had mutations in the flagellar motor genes (*pomA* and *motX*, respectively), whereas the remaining three strains had mutations in the flagellar apparatus (*flhA* and *fliF*) (14) and regulatory genes (*flrC*; Table 1).

**TABLE 1:** Mutations present in the five naturally occurring motility-defective isolates^*a*^

Isolate	Chromosome	Position	Mutation	Annotation	Gene	Function/description
A/20d	I	858,888	C→*T*	Q533* (CAG→TAG)	*flhA*	Flagella export and assembly
B/30d	I	793,656	+GTTA	Coding (83/1434 nt)	*flrC*	Flagellar regulatory
	I	1,463,448	C→A	D119Y (GAT→TAT)	06735	Hypothetical protein
	I	2,915,821	G→A	Intergenic (−135/+157)		
C/20d	I	796,416	Δ1 bp	Coding (1007/1743 nt)	*fliF*	Flagellar M-ring
D/10d	I	2,130,444	Δ2 bp	Coding (493–494/765 nt)	*pomA*	Sodium-type flagellar motor
E/10d	I	311,460	Δ4 bp	Coding (205–208/636 nt)	*motX*	Sodium-type flagellar motor
A, B, C, D, E	I	2,145,828	A→*T*	V33V (GTT→GTA)	*tnpA*	IS1004 family transposase
A, B, D, E	II	13,451	G→A	Intergenic (−138/+28)		
B, C, D	I	487,257	Δ1 bp	Intergenic (+73/+205)		
	I	401,161	C→*T*	Noncoding (188/1543 nt)	01960	16S ribosomal RNA

^*a*^ Isolate names are omitted after the slash in their common mutation sites.

### Motility defects affect culturability of *V. cholerae*

**Fig 3:**
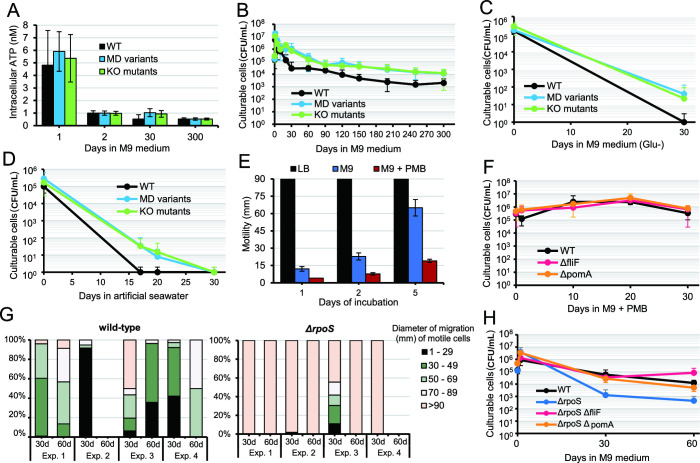
Motility-defective *V. cholerae* strains maintain higher culturability than the motile wild-type strain. (**A**) Intracellular ATP levels in five MD variants (A/20d, B/30d, C/20d, D/10d, and E/10d), five knockout mutants (Δ*flhA*, Δ*flrC*, Δ*fliF*, Δ*pomA*, and Δ*motX*), and WT were measured at days 1, 2, 30, and 300. (**B**) Culturable cell numbers were higher in the MD and KO groups after day 30 than in the WT strain. (**C**) WT and the 10 MD and KO strains were cultured in glucose-free M9 medium for 30 days and (**D**) in artificial seawater. The number of culturable cells was counted by plating on LB agar. (**E**) Polymyxin B (PMB) inhibits the swimming motility of *V. cholerae*. Swimming halos of WT on three motility plates containing either LB, M9, or M9 with 200 µg/mL of PMB (equivalent to 50% minimum inhibitory concentration for the WT strain) were measured during 5 days of incubation. (**F**) WT and the mutants Δ*fliF* and Δ*pomA* were cultured in M9 containing PMB for 30 days. (**G**) Distribution of the diameter of migration in the population of WT and Δ*rpoS* mutant at days 30 and 60. (**H**) Effect of *rpoS* deletion from WT and KO mutants on their culturability. Data represent the mean ± standard deviation of at least three independent experiments.

The five naturally occurring motility-defective (MD) variants and five laboratory-generated knockout (KO) mutants (*ΔflhA, ΔflrC, ΔfliF, ΔpomA*, and *ΔmotX)* (see additional supplemental data [FigShare at DOI: 10.6084/ m9 .figshare.22249498]) were compared with the WT strain in the following experiments. Intracellular ATP levels of WT, MD variants, and KO mutants showed no differences (Fig. 3A). In addition, intracellular ATP levels remained low from day 2 onward. No difference in total cell count was observed. Still, the MD variants and KO mutants showed a higher number of culturable cells than did WT cells on day 30 of culture (Fig. S2; Fig. 3B). Thereafter, WT showed a gradual decrease in culturable cells in parallel with MD mutants, presumably due to impaired or reduced motility (Fig. 1D and E).

The WT population ultimately entered the unculturable state on day 30 in glucose-free M9 medium (no carbon source) and on day 17 in artificial seawater. In contrast, the MD mutants remained culturable for longer periods (Fig. 3C and D), and only a few MD isolates were observed in the WT strain in the artificial seawater on day 14. WGS revealed that all the three isolates we examined had mutations in flagella-related genes (*flhA* and *fliM*), implying that this mutation phenomenon was not restricted to the M9 medium (data not shown).

Next, we observed changes in culturability under conditions in which motility was inhibited by a chemical substance. Subinhibitory concentrations of polymyxin B (PMB) in *V. cholerae* reportedly increase flagellar abnormalities and the number of aflagellated cells (15). PMB (200 µg/mL) was added to M9 motility agar to confirm that the motility of the WT strain was inhibited (Fig. 3E). This concentration of PMB corresponded to half the minimum inhibitory concentration for the WT strain. PMB supplementation allowed WT to maintain higher culturability and show similar levels to those of KO mutants (Δ*filF* and Δ*pomA*) even at day 30 (Fig. 3F). This finding suggests that culturability may be maintained under motility inhibition caused by chemical or physical factors, even in the absence of genetic mutations.

RpoS controls a global adaptive response that allows many Gram-negative bacteria to survive starvation and various stresses (16 – 18). All isolates in the *rpoS* gene-deletion mutant showed motility on day 60 in three of four independent experiments (Fig. S3) and, compared with WT, maintained high motility even after days 30 and 60 (Fig. 3G). The number of culturable cells was lower in the Δ*rpoS* mutant than in the WT (Fig. 3H). The *rpoS/fliF* and *rpoS/pomA* double-KO mutants showed a lower reduction in the number of culturable cells (Fig. 3H). In *E. coli*, expression of genes required for flagellar function, and chemotaxis is elevated in the *rpoS* mutant. The motility of mutants is also enhanced on M63 minimal plates compared with that of WT (19). In contrast, in *V. cholerae*, the *rpoS* mutant showed reduced motility on nutrient-limited plates and decreased expression of related genes compared with those in WT (20, 21). In this study, the *rpoS* mutant showed a shorter migration distance up to 24 hours on M9 motility agar; however, after 48 hours, the migration distance was similar to that of the WT (Fig. S4). Nonetheless, the mechanism by which *rpoS* mutants maintain high motility during long-term incubation remains unknown.

**Fig 4:**
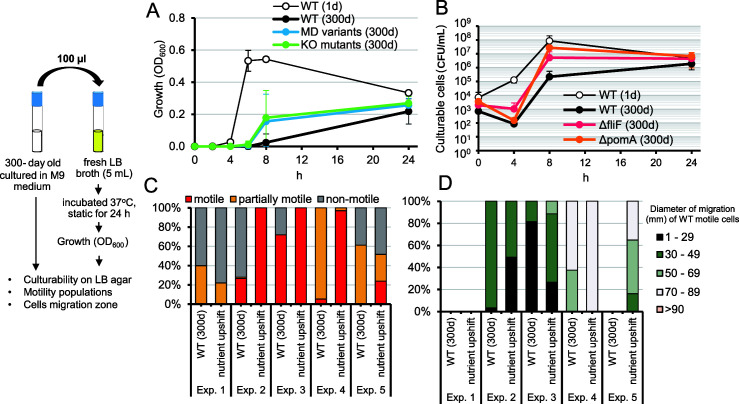
Effects of nutrient upshift on the growth and motility of *V. cholerae* cultured for 300 days. The 300-day old prolonged culture cells were examined for resuscitation by inoculating in 5 mL of fresh LB and incubating at 37°C for 24 hours under static conditions. The effects of nutrient upshift were monitored (**A**) by measuring the optical density at 600 nm of wild-type and motility-defective strains and (**B**) by counting the number of culturable cells among WT and the *fliF* and *pomA* mutants. (**C**) Percentages of motile, partially motile, and non-motile phenotypes, and (**D**) distribution of diameter of migration were measured at day 300 before and after nutrient upshift. Data represent the mean ± standard deviation of five independent experiments.

Next, we analyzed the modulation of growth and motility of the starved WT and MD mutants by adding nutrient sources (LB). Culture media (100 µL) from 300-day old WT, MD variants, and KO mutants were inoculated into fresh LB (5 mL) and incubated at 37°C under static conditions. MD mutants grew faster than the 300-day old WT after 8 hours of incubation (Fig. 4A and B). Nutrient supplementation modulated the motile and motility-impaired populations, altering the migration distances on motility agar (Fig. 4C and D). Experiment 1 showed that the entire population shifted to an MD phenotype, and motile cells were no longer detectable. A complete shift was also observed in cultures of WT and *rpoS* mutants (Fig. 3G), supporting the observation that MD cells maintain high culturability.

### Mutations in flagellar-related genes

**Fig 5:**
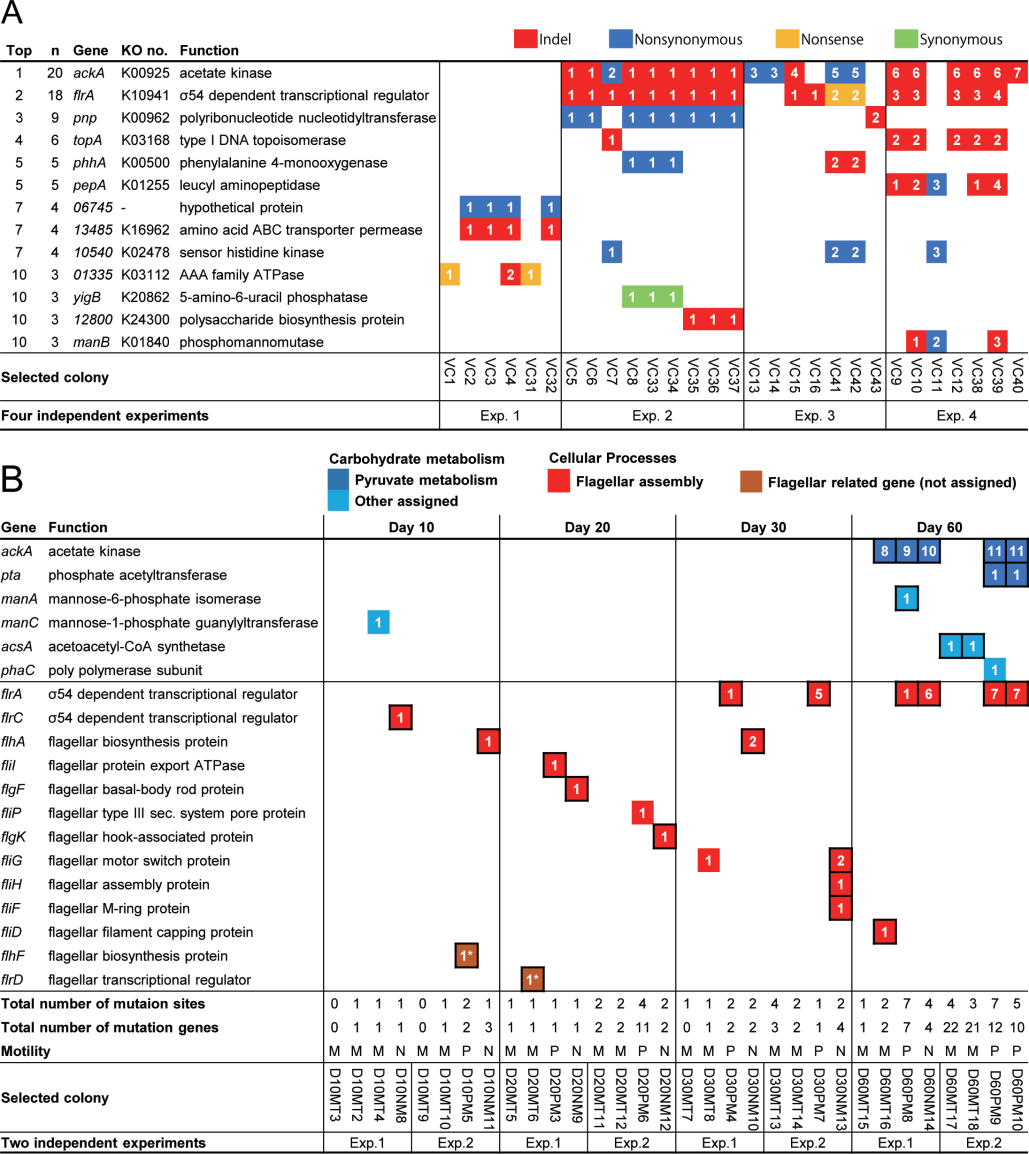
Frequently mutated genes during prolonged incubation. (**A**) Top 10 frequently mutated genes among the 29 isolates on day 60 of prolonged culture. KO number is based on the KEGG database. Numbers in the colored cells represent differences in the mutated sequences of each gene. (**B**) Mutated genes associated with carbohydrate metabolism and flagellar detected among the 32 isolates during 10–60 days of incubation. At each time point, four isolates consisting of two motile and two motility-defective isolates were randomly selected from two independent experiments only if they were present within 100 colonies. Alphabetical abbreviations for motility indicate the following: M, motile; P, partially motile; N, non-motile. Genes framed in black have indel or nonsense mutations. *flhF* belongs to bacterial motility proteins in protein families: signaling and cellular processes. *flrD* was not assigned by BlastKOALA.

In the aforementioned experiments, all five MD variants had mutations in flagella-related genes, but almost all genes and intergenic and noncoding regions of their genomes were conserved (Table 1). Therefore, next, we investigated the pattern of mutation occurrence in long-term cultivation. To increase the probability of obtaining various types of mutant isolates, colonies were randomly selected from the culture on day 60. Then, six phenotypic assays (for colony size, motility, hemolysis, proteolysis, catalase activity, and biofilm formation) were performed for each colony (Table S2). In total, 29 colonies from 4 independent experiments were selected and sequenced. Twenty (69%) of the 29 selected colonies were of MD variants, and the remaining 9 (31%) were motile. In total, 152 mutations, including 142 genes, were detected in the 29 isolates, with an average of 4.9 mutated genes per colony (Table S3). Genes with a high mutation frequency were the acetate kinase gene *ackA* (22) (69.0%) and *flrA* (23) (62.0%), which is a master regulator of the flagellar genes of *V. cholerae* (Fig. 5A). Mutations in flagella-related genes were detected in 22 strains (75.9%; Fig. S5). Next, mutations occurring in the genome of *V. cholerae* were examined over time. Mutations in motility-related genes were detected from day 10 of culture, and by day 60, 18 of the 32 isolates (56.3%) harbored mutations in 13 different flagellar-related genes (Fig. 5B; Table S3). Although isolate D30NM13 had a loss of a DNA region containing three flagellar-related genes, no mutations were detected in multiple flagellar-related genes in all 73 isolates (52 of which showed only one mutation in flagellar-related genes). This may indicate that the goal of reducing motility through mutations was achieved and that mutational pressure on flagellar-related genes had decreased.

Follow-up mutation analysis showed that no *ackA* mutations occurred during the initial 30 days; however, on day 60, five of the eight isolates showed four mutation patterns in *ackA*. These results suggested that mutations occurred in flagellar-related genes in the early phase of prolonged culture in nutrient-limited conditions. In the later stage (after day 30), mutations occurred in *ackA*. Although the biological significance of mutations in *ackA* is unknown, the fact that mutations occur more frequently after day 30 of culture than before may underscore an essential role in the adaptation to environmental changes. In addition, *V. cholerae* has two copies of the acetate kinase gene (53.4% identity of amino acid sequences), and all detected genes harboring mutations were located close to *pta*. The Pta-AckA pathway in *E. coli* is involved in the strong bidirectional exchange of acetic acid with the environment (24). Acetate production by acetate kinase is significant for ATP generation by substrate-level phosphorylation during anaerobic growth in *E. coli* (25). Deletion of *ackA* increased protein acetylation and is involved in the regulation of gene expression (25). Deficiency in acetate kinase may lead to a decrease in the synthesis of acetyl-CoA, which potentially inhibits key energy-generating reactions, such as oxidative phosphorylation and fatty acid synthesis, within the cell. Reducing the diversity of metabolism related to acetate kinase and maintaining or enhancing other metabolic pathways may confer an advantage for bacterial survival (cultivability) under nutrient-depleted conditions.

### Loss of genomic integrity with prolonged culture

**Fig 6:**
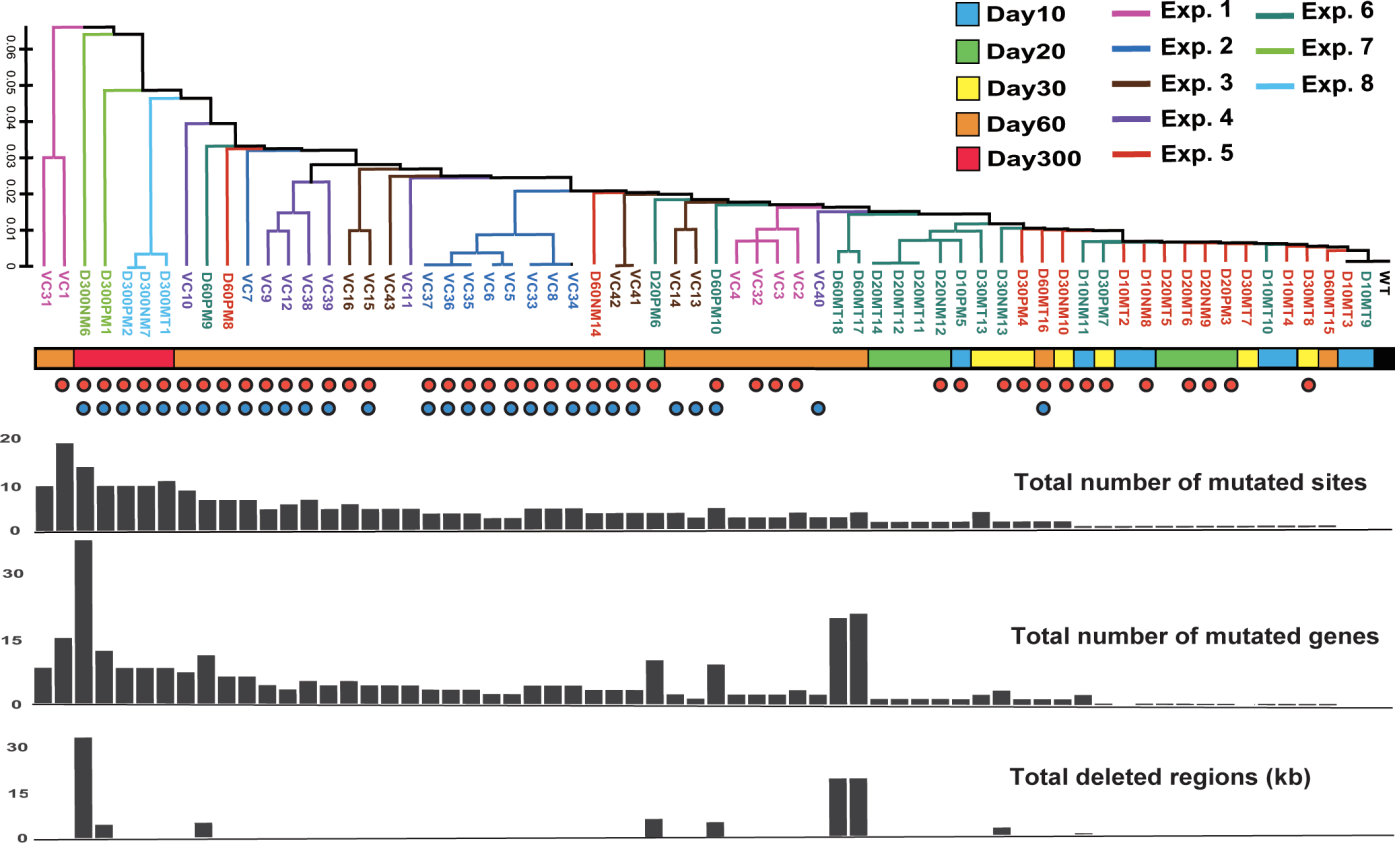
Genetic relatedness and mutation profile of the isolates derived from wild-type MS84A after prolonged culture. Dendrogram of the 66 isolates and WT was generated via UPGMA cluster analysis using alignment data obtained by the “gdtools” command packaged with “breseq.” Each independent experiment and culture period are color coded. Red and blue dots indicate mutations in the flagella-related genes and *ackA*, respectively. Values of bars shown at the bottom of the tree correspond to the results for each isolate.

WGS was performed on five isolates of *V. cholerae*, cultured for up to 300 days. A total of 54 mutations, including 79 genes, were detected in the five isolates, with an average of 15.8 mutated genes per isolate (Table S3). Mutations in flagella-related genes and *ackA* were detected in all five isolates. Mutated regions from the 66 genomes of the isolates derived from WT were aligned. A phylogenetic tree was constructed using the UPGMA method (Fig. 6). These variants tended to form clusters in each independent experiment. Exceptions were VC1 and VC31, which diverged from the main cluster, with both isolates harboring non-synonymous substitutions in *mutS*. MutS is a mismatch DNA repair protein (26). Therefore, we constructed a *mutS* deletion mutant and investigated its mutations. In the two randomly selected isolates from two independent experiments, 50 mutations, including 46 genes, were detected, with an average of 23 mutated genes per isolate, suggesting that *mutS* deletion in *V. cholerae* increased genomic variation (Table S3). The two *mutS* mutants at 60 days of age also had mutations in both *flrA* and *ackA*.

Out of the 73 isolates (including *mutS* mutants), 10 showed a loss of DNA regions containing multiple genes (see additional supplemental data [FigShare at DOI: 10.6084 /m9 .figshare.22249498]). In addition, two deletions of regions containing motility-related genes and three deletions of *ackA*-containing regions, the loss of virulence factors, including CTX phage, and the loss of large regions (up to 35 kb) were observed. The variants that produced little or no cholera toxin owing to the loss of CTX phage or other functions were detected (Fig. S6).

### Prolonged survival of *V. cholerae*

Flagella-mediated motility is intimately connected to biological and cellular processes, such as chemotaxis, biofilm formation, colonization, and virulence of *Vibrio* spp. (27, 28). However, it is expensive to assemble and energize flagella for motility, and this can become a burden when energy sources are scarce (29, 30). Thus, it is reasonable that under prolonged nutrient deprivation, bacteria would decrease or stop their motility to maintain cellular stability. In this study, we observed the transition of *V. cholerae* to the MD phenotype after long-term incubation under nutrient deprivation; furthermore, these MD mutants maintained higher culturability than the motile strains, including the WT strain and *rpoS* mutants. Maintenance of culturability leads to a rapid population growth of *V. cholerae* when exposed to nutrient sources compared with that in VNC cells, resulting in its long-term survival in the environment. However, if culturability is maintained long term, the loss of motility, virulence factors, and various metabolic functions occurs, compromising genomic integrity. The resulting variants might reduce the ability to adapt to diverse natural environments. Regarding the drawbacks of mutational changes, halting DNA replication in the VNC state may affect the preservation of their genomic integrity (31, 32). The VNC state is an inactive life form awaiting resuscitation under appropriate conditions (7 – 10, 33). We found that more colonies grew on sheep blood agar than on LB agar, suggesting that sheep blood restores the growth ability of some populations in the VNC state after prolonged culture in the M9 medium (see additional supplemental data [FigShare at DOI: 10.6084 /m9 .figshare.22249498]). Thus, *V. cholerae* in the VNC state may regain their ability to multiply and infect humans when exposed to blood spills in slaughterhouses and during meat washing.

Prolonged survival under environmental stress in long-term batch cultures can lead to the selection of mutants with a growth advantage in the stationary phase “GASP” (34, 35). A previous study described that GASP *V. cholerae* O1 persisted in nutrient-poor lake water microcosms for 700 days, showing decreased motility, increased VPS-independent biofilm formation, and oxidative stress resistance when grown in filter-sterilized lake water, compared with the corresponding WT strain (36). Recently, Gao et al. (37) reported that a series of stress resistance-related genes were upregulated in the non-O1/O139 *V. cholerae* starved for 6 months, concomitant with the downregulation of flagellum assembly-related genes compared with non-starved control bacteria. Under our condition, impaired or reduced motility variants with mutations in *ackA* had become predominant in culturable cells during the stationary phase. The genomic and phenotypic variations may be caused by a survival mechanism, whereby *V. cholerae* adapts to its environmental niche by accumulating mutations.

The present study has a few limitations. Our investigation was conducted in the M9 medium under the following conditions: static, 37°C, and pH 7.0–7.5, which did not change during long-term cultivation. Furthermore, the results obtained were based on free-floating cells with no cell aggregation. Because human-infecting *V. cholerae* forms biofilm-like aggregates (38), cells that have physically ceased motility and cells that have entered a VNC state owing to environmental stresses inside and outside the intestinal tract may coexist in aggregates. It remains to be determined whether complex cell aggregates can maintain genomic integrity and culturability for a long duration.

The VNC cells that emerged in the early phase of prolonged culture appear to transition to an inactivated state under starvation stress. Their emergence can be partially suppressed by reducing energy-requiring motility, and most VNC cells did not revert to culturable cells (heterogeneous population). It is unlikely that such VNC cells proactively influence mutations toward being culturable cells. The findings of this study provide insights into the dynamics of microbial populations in long-term cultures and highlight the need for further investigation into the role of VNC cells in microbial ecology. Our future research will explore the interaction between VNC cells and culturable cells.

## MATERIALS AND METHODS

### WT strain

The toxigenic *V. cholerae* strain MS84A (O1, El Tor, serotype Ogawa) was isolated in Thailand in 2010 from a patient with diarrhea (39) and was used as the wild-type (WT) strain in this study. The complete genome sequence of the strain was determined. In brief, Unicycler (40) (v.0.4.8) was used for the hybrid assembly of sequence reads from the Illumina MiSeq system (300 bp paired-end sequencing) and from Oxford Nanopore MinION sequencing. The BUSCO (41) score was 99.8% (the ratio of the number of genes found in the assembly out of 1,445 Vibrionales core genes), suggesting that the genome assembly was of high quality. The National Center for Biotechnology Information (NCBI) Prokaryotic Genome Annotation Pipeline (PGAP) annotation (42) indicated 2,570 and 1,003 coding DNA sequences in chromosomes 1 (2,962,973 bp) and 2 (1,044,173 bp), respectively.

### Culture media and growth conditions

Luria–Bertani (LB) broth containing (per liter) 10 g tryptone, 5 g yeast extract, and 10 g NaCl was used as a nutritionally rich medium in this study. The bacterial stock was stored at −80°C in LB containing 25% (vol/vol) glycerol. A single colony of each bacterium on an LB agar plate was cultured in LB with shaking (120 rpm) at 37°C overnight for use in subsequent experiments.

For prolonged culture, the M9 medium, consisting of 2 g/L glucose (0.2%), 0.1 mM CaCl_2_, 2.0 mM MgSO_4_, and M9 minimal salts (Difco), was used. Overnight bacterial cultures grown at 37°C in LB were centrifuged at 12,000 × *g* for 1 minute and washed twice with an equal volume of phosphate‐buffered saline (PBS) (Sigma). Thereafter, the cells were resuspended in PBS to the original culture volume. Bacterial suspensions (25 µL) were transferred into 14-mL polystyrene tubes containing 5 mL of M9, M9 (0% glucose), or artificial seawater (40 g/L sea salt; Sigma) and incubated at 37°C under static conditions. Sterile water was added only to compensate for evaporation.

### Bacterial counts

The total number of cells in the culture was counted using an SLGC bacteria counter (Minato Medical, Tokyo, Japan). The number of culturable cells (colony-forming units) was determined by dilution plating on LB agar and incubating at 37°C overnight.

Cell viability was determined using the LIVE/DEAD BacLight kit (L13152; Invitrogen), according to the manufacturer’s instructions. From each bacterial suspension, a 500-µL aliquot was centrifuged at 12,000 × *g* for 1 min, and the pellet was resuspended in 125 µL PBS. The cells were stained with a 125-µL mixture (1:1) of SYTO 9 and propidium iodide and incubated in the dark for 20 minutes. The cells were placed on glass slides and examined with a 60× objective lens using a Nikon Eclipse Ti confocal fluorescence microscope (Nikon Instrument Inc.). Images were captured using NIS-Elements AR software version 4.11.00 (Nikon Instrument Inc.). For each sample, 12 random fields (at least 15,000 cells in each independent experiment) were visualized. Live cells were stained green, whereas dead cells were stained red. The green and red images were counted using the ImageJ software (v.1.52a) (http://rsb.info.nih.gov/ij/). The percentage of viable cells was calculated as follows: % live cells = [live cell count (green cells)/total cell count (green cells + red cells)] × 100.

### Motility assay

Colonies grown on LB agar were picked up using sterile toothpicks, stabbed on motility agar plates (LB or M9 broth media containing 0.3% agar), and incubated face up at 37°C for 24 hours. Swimming proficiency was determined by measuring the diameter of the halo on motility agar plates. The non-motile phenotype was embedded at the point of inoculation, whereas the partially motile displayed a star-like penetration pattern.

### Visualization of flagella using transmission electron microscopy

An overnight culture in LB broth was washed and diluted in PBS to yield an OD_600_ of 0.3. Aliquots of a 100-µL drop of each diluted culture were adsorbed onto Formvar-coated grids for 2 minutes, and the excess fluid was removed with a filter paper. After 2 minutes, each grid was washed gently with distilled water and negatively stained with 2% (wt/vol) aqueous uranyl acetate solution for 1 minute; the excess fluid was removed with a filter paper, and the grid was air dried. The cells were examined using a Hitachi HT77000 transmission electron microscope.

### Construction of gene deletion mutants

The construction of deletion mutants of *V. cholerae* was performed via double-crossover homologous recombination. In brief, fragments were constructed by amplifying approximately 800 bp regions upstream and downstream of the targeted deletion region and were cloned into the R6K-ori suicide vector, pYAK1 (43), which contains a chloramphenicol resistance cassette and the counter-selectable marker *sacB*, at the *Bam*HI and *Pst*I restriction sites using Gibson assembly Master Mix (New England BioLabs), according to the manufacturer’s instructions. The *Escherichia coli* SM10 λpir was used as the conjugative donor strain. For plasmid maintenance in *E. coli* and *V. cholerae*, chloramphenicol was added to the media at concentrations of 25 and 5 µg/mL, respectively. Conjugants were selected on thiosulfate citrate bile salts sucrose agar plates containing chloramphenicol and passed through three passages in LB containing 10% sucrose in the absence of antibiotics. Finally, sucrose-resistant/chloramphenicol-sensitive colonies were screened using polymerase chain reaction (PCR). The deletion regions were confirmed by DNA sequencing using the ABI 3130xl sequencer and BigDye Terminator v3.1 (Applied Biosystems). Primer sequences used for PCR and sequencing are available in the additional supplemental data (FigShare at DOI: 10.6084 /m9 .figshare.22249498).

### Determination of intracellular ATP levels

One milliliter of cell culture was harvested and washed with PBS. The cells were extracted with 100 µL of 1% trichloroacetic acid buffer at 4°C for 10  minutes, and 900 µL of Tris-acetate buffer pH 7.75 was added before mixing with the rL/L reagent (ENLITEN ATP assay, Promega) in accordance with the manufacturer’s instructions. Emitted luminescence was detected using a Centro LB960 luminometer (Berthold Technology). A standard curve was generated from known concentrations of ATP and used to calculate intracellular ATP levels.

### Genome sequencing and mutation analysis

Genomes were extracted from *V. cholerae* isolates using the DNeasy Blood & Tissue Kits (Qiagen), according to the manufacturer’s instructions. The genomes of the five derived isolates described in Table 1 were sequenced on the Ion Torrent PGM system (Life Technologies) following the manufacturer’s protocols for 400 bp genomic DNA (gDNA) fragment library construction, template preparation, and sequencing (Ion PGM Hi-Q view chef 400 kit). The genomes of the other derived isolates, shown in Table S3, were sequenced on the Illumina MiSeq platform. DNA library preparation was performed with 500  ng of gDNA extract using the Illumina DNA Prep kit (Illumina, San Diego, CA, USA) and Nextera DNA CD Indexes (96 Indexes) (Illumina), according to the manufacturer’s instructions. The 20 pM final pool libraries were loaded into a 600-cycle v3 MiSeq reagent cartridge. For preprocessing of Illumina reads, Fastp (v.0.20.1) (44) was used to detect and remove adapters and bases with a Phred quality score below 30. All sequence data of the 73 derived isolates were deposited in GenBank under bioproject no. PRJNA714234.

To identify mutations in the genomes, we used breseq (v.0.35.7) (45), which uses Bowtie2 (46), to map sequence reads to the reference genome. In addition, we used the gdtools in the breseq software to obtain alignment data of mutations among the derived isolates and the reference. A dendrogram was constructed using the unweighted pair group method with arithmetic mean (UPGMA) method of MEGA (v.11.0.11) (47). Protein sequences from genes with detected mutations were subjected to functional annotation using the Kyoto Encyclopedia of Genes and Genomes (KEGG) database (48) using BlastKOALA (https://www.kegg.jp/blastkoala/).

## Data Availability

The complete genome sequence of the wild-type strain of *V. cholerae* O1 was deposited in GenBank (accession numbers CP077060-CP077061). Shotgun sequencing data supporting the findings of this study have been deposited in GenBank under accession code PRJNA714234. Additional supplemental data are available in FigShare at DOI: 10.6084/m9.figshare.22249498.

## SUPPLEMENTAL MATERIAL

Fig. S1 msystems.00109-23-s0001.pdf Reversion of *Vibrio cholerae* from the motility-defective form to the motile form.

Fig. S2 msystems.00109-23-s0002.pdf Cell number of motility-defective *V. cholerae* during long-term incubation.

Fig. S3 msystems.00109-23-s0003.pdf Percentages of motile, partially motile, and non-motile phenotypes.

Fig. S4 msystems.00109-23-s0004.pdf Effect of *rpoS* deletion on swimming motility.

Fig. S5 msystems.00109-23-s0005.pdf Mutated genes belong to the same groups of KEGG pathway function as *ackA* and *flrA*.

Fig. S6 msystems.00109-23-s0006.pdf Cholera toxin production in the isolates derived from MS84A after long-term culture.

Table S1 msystems.00109-23-s0007.pdf (A) Frequency of reversion from the partially motile form to the motile form. (B) Frequency of reversion from the non-motile form to the motile form.

Table S2 msystems.00109-23-s0008.pdf (A) Criteria for classification to distinguish colonies. (B) Profiles of colonies classified and selected for whole genome sequencing.

Table S3 msystems.00109-23-s0009.pdf Mutation profiles of the 68 isolates derived from *V. cholerae* O1 MS84A.

Supplemental Text msystems.00109-23-s0010.doc Supplemental methods.

ASM does not own the copyrights to Supplemental Material that may be linked to, or accessed through, an article. The authors have granted ASM a non-exclusive, world-wide license to publish the Supplemental Material files. Please contact the corresponding author directly for reuse.
