# Supplementary material for: Flagella-related gene mutations in Vibrio cholerae during extended cultivation in nutrient-limited media impair cell motility and prolong culturability
Source: mSystems. 2023 Aug 29;8(5):e00109-23. doi: 10.1128/msystems.00109-23 (PMC10654082; doi:10.1128/msystems.00109-23)
Supplement: Fig. S1 — Reversion of Vibrio cholerae from the motility-defective form to the motile form. [file msystems.00109-23-s0001.pdf]

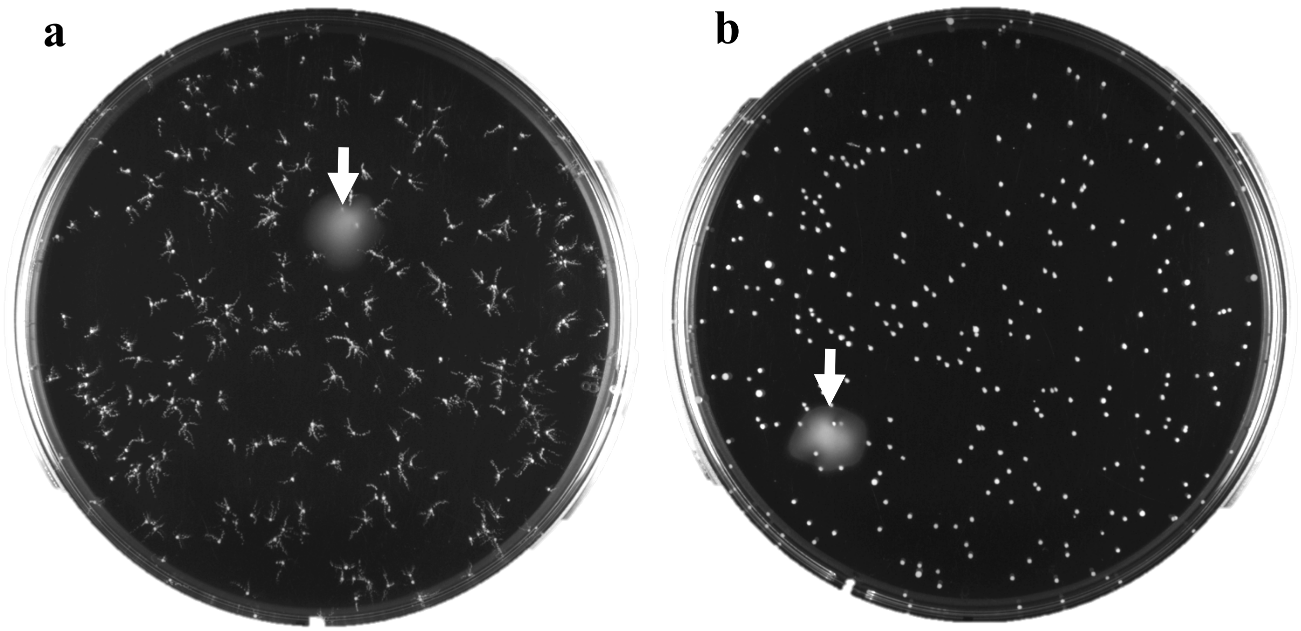

Fig. S1. Reversion of *Vibrio cholerae* from the motility-defective form to the motile form. Motile revertants were observed on motility agar plates after 24 h of incubation at 37°C. **a**, The arrow indicates a partially motile cell that reverts to a motile cell. **b**, The arrow indicates that a non-motile cell reverted to a motile cell. The frequencies of reversion from the motility-defective to motile form are shown in Table S1.
