# Supplementary material for: Flagella-related gene mutations in Vibrio cholerae during extended cultivation in nutrient-limited media impair cell motility and prolong culturability
Source: mSystems. 2023 Aug 29;8(5):e00109-23. doi: 10.1128/msystems.00109-23 (PMC10654082; doi:10.1128/msystems.00109-23)
Supplement: Fig. S2 — Cell number of motility-defective V. cholerae during long-term incubation. [file msystems.00109-23-s0002.pdf]

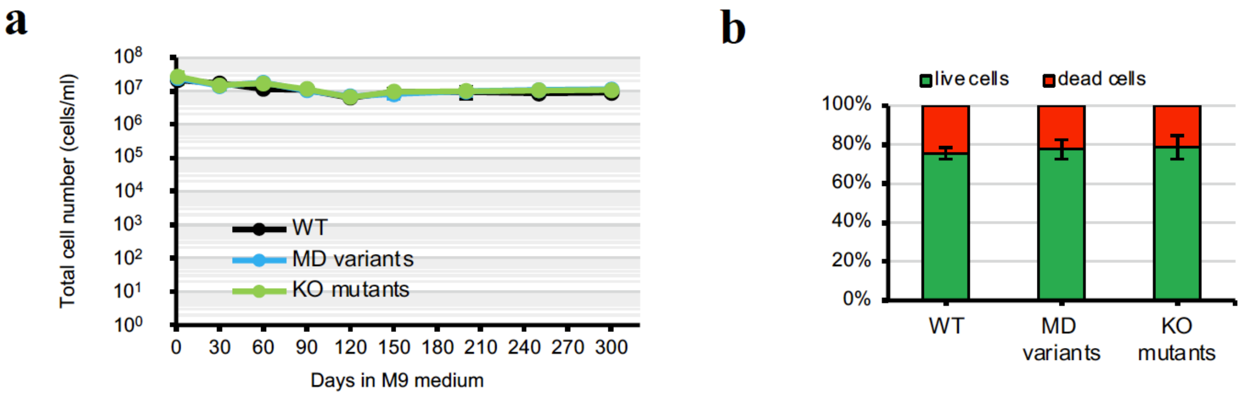

Fig. S2. Cell number of motility-defective *V. cholerae* during long-term incubation. a, Number of total cells and **b**, live/dead cells of wild-type, five naturally occurring motility-defective (MD) variants (A/20d, B/30d, C/20d, D/10d, and E/10d), and five knockout (KO) mutants (Δ*flhA*, Δ*flrC*, Δ*fliF*, Δ*pomA*, and Δ*motX*) was counted during prolonged incubation in M9 minimum medium at 37°C. The total cell numbers of all 10 mutants were approximately 10^7, and the percentages of live and dead cells on day 30 showed no difference.
