# Supplementary material for: Flagella-related gene mutations in Vibrio cholerae during extended cultivation in nutrient-limited media impair cell motility and prolong culturability
Source: mSystems. 2023 Aug 29;8(5):e00109-23. doi: 10.1128/msystems.00109-23 (PMC10654082; doi:10.1128/msystems.00109-23)
Supplement: Fig. S3 — Percentages of motile, partially motile, and non-motile phenotypes. [file msystems.00109-23-s0003.pdf]

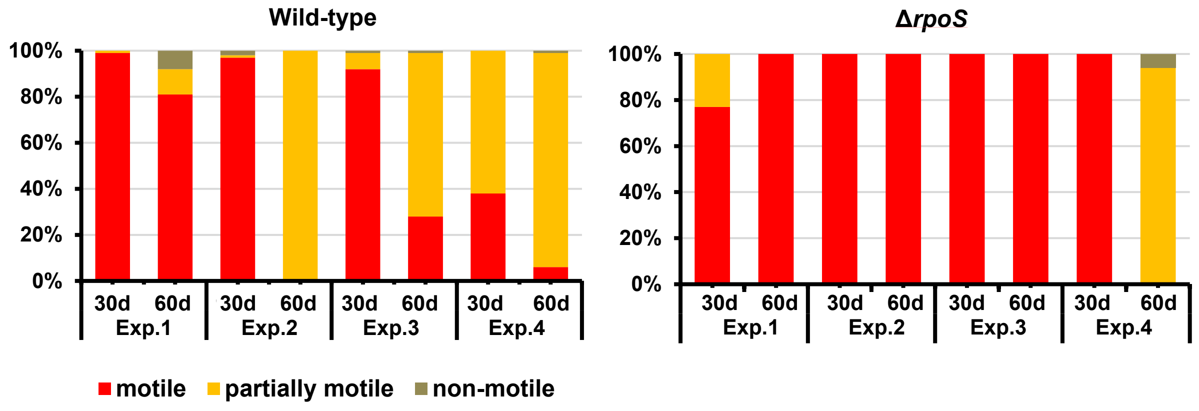

Fig. S3. Percentages of motile, partially motile, and non-motile phenotypes. The percentages of motile, partially motile, and non-motile cells were determined using at least 100 randomly selected colonies each of WT and *rpoS*-deletion mutant at days 30 and 60 from four independent experiments.
