# Supplementary material for: Flagella-related gene mutations in Vibrio cholerae during extended cultivation in nutrient-limited media impair cell motility and prolong culturability
Source: mSystems. 2023 Aug 29;8(5):e00109-23. doi: 10.1128/msystems.00109-23 (PMC10654082; doi:10.1128/msystems.00109-23)
Supplement: Fig. S4 — Effect of rpoS deletion on swimming motility. [file msystems.00109-23-s0004.pdf]

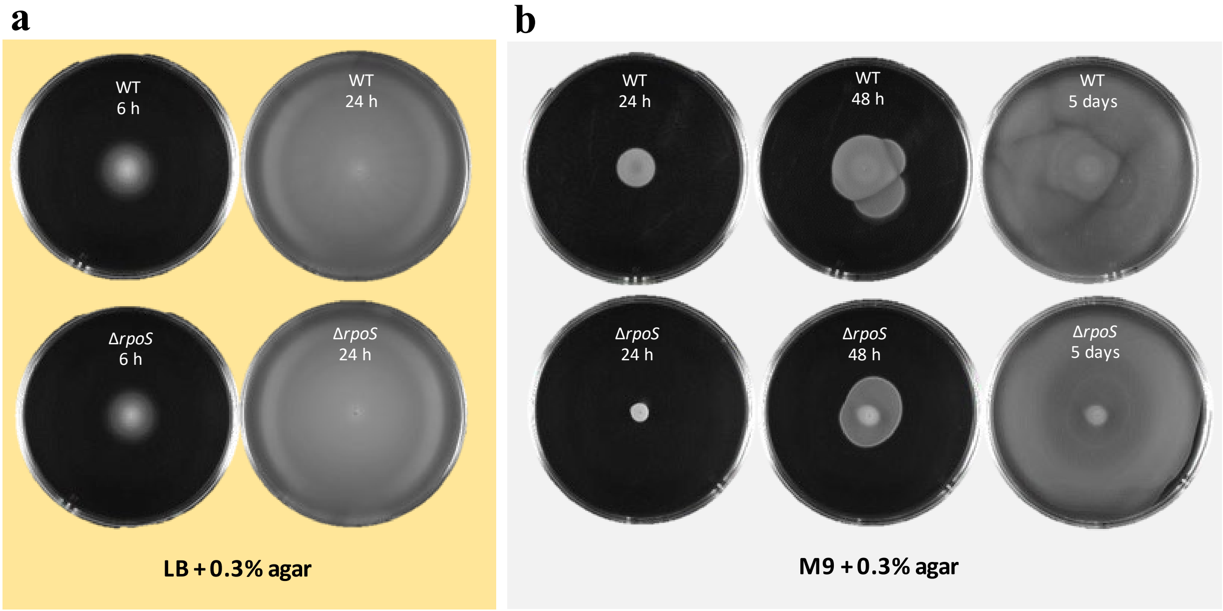

Fig. S4. Effect of *rpoS* deletion on swimming motility. Motility of *V. cholerae* wild-type and the *rpoS* gene knockout mutant (*ΔrpoS*) in **a**, LB containing 0.3% agar and **b**, M9 containing 0.3% agar after incubation at 37 ^{o}C. The motility of the WT and the *rpoS* mutant showed similar levels on LB agar at two time points (6 and 24 h), whereas the *rpoS* mutant showed less motility during the first 24 h on M9 agar; however, at later time points (48 h and 5 days), WT and the *rpoS* mutant showed similar motility.
