# Supplementary material for: Flagella-related gene mutations in Vibrio cholerae during extended cultivation in nutrient-limited media impair cell motility and prolong culturability
Source: mSystems. 2023 Aug 29;8(5):e00109-23. doi: 10.1128/msystems.00109-23 (PMC10654082; doi:10.1128/msystems.00109-23)
Supplement: Fig. S5 — Mutated genes belong to the same groups of KEGG pathway function as ackA and flrA. [file msystems.00109-23-s0005.pdf]

Carbohydrate metabolism: Pyruvate metabolism (blue)
Cellular Processes: Flagellar assembly (red); Biofilm formation (green)
Protein families: genetic information processing: Transcription factors (yellow)
Environmental Information Processing: Two-component system (purple)

Category	Gene	Function	VC1	VC2	VC3	VC4	VC31	VC32	VC5	VC6	VC7	VC8	VC33	VC34	VC35	VC36	VC37	VC13	VC14	VC15	VC16	VC41	VC42	VC43	VC9	VC10	VC11	VC12	VC38	VC39	VC40
Pyruvate metabolism	***ackA***	**acetate kinase**							1	1	2		1	1	1	1	1	3	3	4		5	5		6	6		6	6	6	7
Pyruvate metabolism	*acs*	acetate–CoA ligase																1													
Pyruvate metabolism	*pckA*	phosphoenolpyruvate carboxykinase (ATP)																	1					2							
Pyruvate metabolism	*pyk*	pyruvate kinase	1				1																								
Flagellar assembly	***flrA***	**σ54 dependent transcriptional regulator**							1	1	1	1	1	1	1	1	1			1	1	2	2		3	3		3	3	4	
Flagellar assembly	*fliF*	flagellar M-ring protein FliF	1					2																							
Flagellar assembly	*flgL*	flagellar hook-associated protein FlgL		1																											
Flagellar assembly	*flhA*	flagellar biosynthesis protein FlhA			1																										
Biofilm formation	***flrA***	**σ54 dependent transcriptional regulator**							1	1	1	1	1	1	1	1	1			1	1	2	2		3	3		3	3	4	
Biofilm formation	*tcpC*	toxin-coregulated pilus secretin TcpC	1																												
Biofilm formation	*dksA*	RNA polymerase-binding protein DksA																									1				
Biofilm formation	*ompT*	porin OmpT																		1	1										
Transcription factors	***flrA***	**σ54 dependent transcriptional regulator**							1	1	1	1	1	1	1	1	1			1	1	2	2		3	3		3	3	4	
Transcription factors	*cysB*	HTH-type transcriptional regulator																		1	1										
Transcription factors	*06390*	LysR family transcriptional regulator																													1
Transcription factors	*dksA*	RNA polymerase-binding protein DksA																		1	1										
Two-component system	***flrA***	**σ54 dependent transcriptional regulator**							1	1	1	1	1	1	1	1	1			1	1	2	2		3	3		3	3	4	
Two-component system	*phoR*	phosphate regulon sensor histidine kinase																							1						
	**Total number of mutation sites**		19	4	3	3	10	3	3	3	7	5	5	5	4	4	4	3	4	5	6	4	4	5	5	9	5	5	7	5	3
	**Total number of mutation genes**		16	4	3	3	9	3	3	3	7	5	5	5	4	4	4	2	3	5	6	4	4	5	5	8	5	3	6	5	3
	**Motility**		M	P	N	M	M	N	P	P	P	P	P	P	P	P	P	M	M	P	P	P	P	P	P	P	M	M	P	M	M
	**Four independent experiments**		Exp. 1						Exp. 2									Exp. 3							Exp. 4						

Fig. S5. Mutated genes belong to the same groups of KEGG pathway function as *ackA* and *flrA*. The numbers in the colored cells represent the differences among the mutated sequences of each gene. Alphabetical abbreviations for motility are as follows: M, motile; P, partially motile; and N, non-motile.
