# Supplementary material for: Flagella-related gene mutations in Vibrio cholerae during extended cultivation in nutrient-limited media impair cell motility and prolong culturability
Source: mSystems. 2023 Aug 29;8(5):e00109-23. doi: 10.1128/msystems.00109-23 (PMC10654082; doi:10.1128/msystems.00109-23)
Supplement: Fig. S6 — Cholera toxin production in the isolates derived from MS84A after long-term culture. [file msystems.00109-23-s0006.pdf]

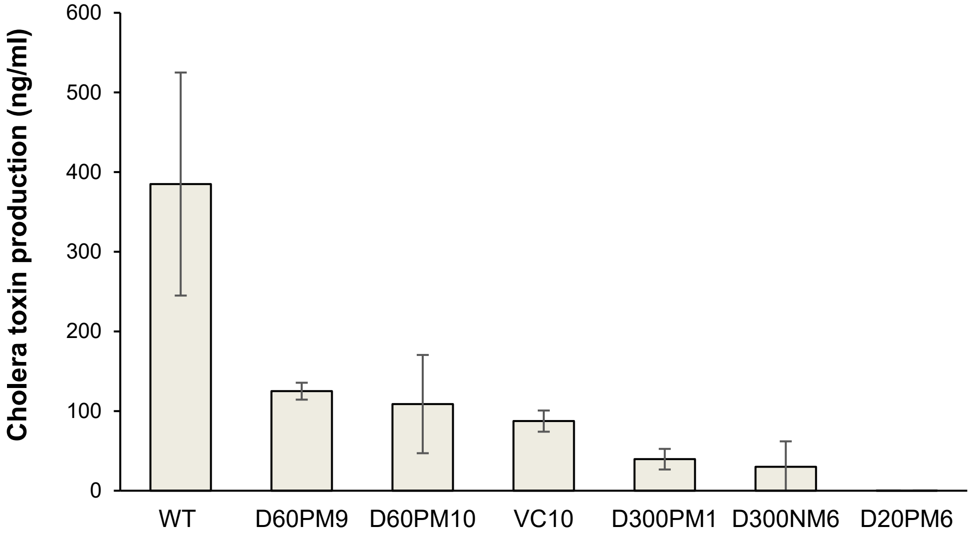

Fig. S6. Cholera toxin production in the isolates derived from MS84A after long-term culture. The results are shown only for strains with significantly lower CT production than that in WT. Isolate D20PM6, which lost the CTX phage encoded by the cholera toxin gene, did not produce the cholera toxin.
