# Supplementary material for: Flagella-related gene mutations in Vibrio cholerae during extended cultivation in nutrient-limited media impair cell motility and prolong culturability
Source: mSystems. 2023 Aug 29;8(5):e00109-23. doi: 10.1128/msystems.00109-23 (PMC10654082; doi:10.1128/msystems.00109-23)
Supplement: Table S1 — (A) Frequency of reversion from the partially motile form to the motile form. (B) Frequency of reversion from the non-motile form to the motile form. [file msystems.00109-23-s0007.pdf]

Table S1.

a | Frequency of reversion from the partially motile form to the motile form

Experiment #	Days of incubation	Colony ID	Number of tested clones	Number of clones regaining motility	Frequency of reversion (%)
1st	20	1.4.4	6,579	32	0.49
1st	20	1.4.5	6,538	44	0.67
2nd	30	2.1.7	4,963	12	0.24
2nd	30	2.1.8	2,642	8	0.3
2nd	30	2.1.9	2,557	37	1.45
2nd	30	2.2.1	3,366	30	0.89
2nd	20	1.8.4	6,381	0	0
2nd	20	1.8.5	7,471	32	0.43
2nd	30	2.4.4	5,059	0	0
2nd	30	2.4.5	5,749	0	0
2nd	30	2.4.6	4,966	0	0
2nd	30	2.4.7	4,799	0	0
2nd	30	2.4.8	4,328	0	0
3rd	20	2.6.8	3,739	7	0.19
6th	10	2.8.8	4,306	1	0.02
7th	5	2.9.3	4,306	0	0
9th	10	2.9.6	3,595	2	0.06
10th	10	3.1.6	6,003	0	0
10th	30	3.2.7	7,947	11	0.14

b | Frequency of reversion from the non-motile form to the motile form

Experiment #	Days of incubation	Colony ID	Number of tested clones	Number of clones regaining motility	Frequency of reversion (%)
1st	20	1.4.3 *	12,246	0	0
2nd	10	1.7.6	5,654	22	0.39
2nd	20	1.8.6	2,937	0	0
2nd	20	1.8.7	2,632	2	0.08
2nd	20	1.8.8	2,791	0	0
2nd	30	2.4.9 †	11,029	0	0
2nd	30	2.5.1	2,159	0	0
2nd	30	2.5.2	2,597	0	0
3rd	20	2.6.4 ‡	15,108	0	0
3rd	20	2.6.5	3,264	1	0.03
3rd	20	2.6.6	3,183	0	0
3rd	20	2.6.7	3,005	2	0.07
3rd	30	2.7.8	4,242	0	0
3rd	30	2.7.9	4,051	0	0
5th	10	2.8.6 §	12,437	0	0
6th	10	2.8.9 ¶	13,722	0	0
6th	10	2.9.1	5,063	0	0
6th	20	2.9.2	4,382	0	0
7th	30	2.9.5	3,686	0	0
7th	10	2.9.4	4,917	0	0
8th	30	3.1.2	3,376	6	0.18
9th	10	2.9.7	2,811	0	0
9th	10	2.9.8	4,480	0	0
9th	10	2.9.9	3,786	0	0
9th	10	3.1.1	3,856	0	0

10th	5	3.1.3	2,685	1	0.04
10th	5	3.1.4	1,706	0	0
10th	5	3.1.5	2,510	0	0
10th	10	3.1.7	2,703	2	0.07
10th	10	3.1.8	2,867	0	0
10th	10	3.1.9	2,819	3	0.11
10th	20	3.2.1	2,752	0	0
10th	20	3.2.2	3,519	0	0
10th	30	3.2.3	2,897	0	0
10th	30	3.2.4	3,347	0	0
10th	30	3.2.5	2,746	0	0
10th	30	3.2.6	2,707	1	0.04

*, A/20d; †, B/30d; ‡, C/20d; §, D/10d; and ¶, E/10d variants
