# Supplementary material for: Flagella-related gene mutations in Vibrio cholerae during extended cultivation in nutrient-limited media impair cell motility and prolong culturability
Source: mSystems. 2023 Aug 29;8(5):e00109-23. doi: 10.1128/msystems.00109-23 (PMC10654082; doi:10.1128/msystems.00109-23)
Supplement: Table S2 — (A) Criteria for classification to distinguish colonies. (B) Profiles of colonies classified and selected for whole genome sequencing. [file msystems.00109-23-s0008.pdf]

Table S2

a | Criteria for classification to distinguish colonies

No.	Morphological/Phenotypical characterization	Reference range for distinguishing colonies				
		1+	2+	3+	4+	5+
1	Colony size	Very small	Small	Normal	Large	
2	Motility (mm)	0	Partial	<40	40–90	>90
3	Hemolysis (mm)	0	1.0–5.5	5.6–7.5	>7.5	
4	Proteolysis (mm)	<3.5	3.5–6.5	>6.5		
5	Catalase activity (units/mg)	<1.9	2.0–3.9	4.0–5.9		
6	Biofilm formation (OD595)	<0.5	0.5–1.5	1.6–3.0		

b | Profiles of colonies classified and selected for whole genome sequencing

Experiment no.	Incubation day(s)	Number of colonies	Phenotypic ID*	Colony size	Motility	Hemolysis	Proteolysis	Catalase	Biofilm	Representative isolate
Reference	Day 1	-	1	3+	5+	2+	3+	1+	2+	WT
Exp.1	Day 60	22	6	3+	4+	2+	3+	1+	2+	VC1
Exp.1	Day 60	3	7	3+	1+	2+	3+	1+	2+	VC32
Exp.1	Day 60	2	11	3+	2+	2+	3+	1+	2+	VC2
Exp.1	Day 60	1	8	3+	1+	2+	3+	2+	2+	VC3
Exp.1	Day 60	1	5	3+	4+	2+	3+	1+	1+	VC4
Exp.1	Day 60	1	6	3+	4+	2+	3+	1+	2+	VC31
Exp.2	Day 60	14	10	3+	2+	2+	3+	1+	1+	VC33
Exp.2	Day 60	5	11	3+	2+	2+	3+	1+	2+	VC5
Exp.2	Day 60	4	16	3+	2+	3+	3+	1+	2+	VC36
Exp.2	Day 60	2	15	3+	2+	3+	3+	1+	1+	VC34
Exp.2	Day 60	1	9	3+	2+	2+	3+	1+	3+	VC6
Exp.2	Day 60	1	12	3+	2+	2+	3+	2+	1+	VC7
Exp.2	Day 60	1	13	3+	2+	2+	2+	1+	1+	VC35
Exp.2	Day 60	1	17	3+	2+	3+	3+	2+	1+	VC37
Exp.2	Day 60	1	18	3+	2+	3+	3+	2+	2+	VC8
Exp.3	Day 60	8	2	3+	3+	2+	3+	1+	1+	VC39
Exp.3	Day 60	7	11	3+	2+	2+	3+	1+	2+	VC10
Exp.3	Day 60	6	10	3+	2+	2+	3+	1+	1+	VC38
Exp.3	Day 60	4	9	3+	2+	2+	3+	1+	3+	VC9
Exp.3	Day 60	2	4	3+	3+	1+	3+	1+	2+	VC11
Exp.3	Day 60	1	3	3+	3+	2+	3+	1+	2+	VC12
Exp.3	Day 60	1	6	3+	4+	2+	3+	1+	2+	VC40
Exp.4	Day 60	11	19	3+	2+	1+	3+	1+	1+	VC15
Exp.4	Day 60	6	11	3+	2+	2+	3+	1+	2+	VC42
Exp.4	Day 60	4	20	3+	2+	1+	2+	1+	1+	VC16
Exp.4	Day 60	3	10	3+	2+	2+	3+	1+	1+	VC41
Exp.4	Day 60	3	14	3+	2+	2+	2+	1+	2+	VC43
Exp.4	Day 60	2	6	3+	4+	2+	3+	1+	2+	VC14

Exp.4	Day 60	1	5	3+	4+	2+	3+	1+	1+	VC13

*Phenotypic IDs show different phenotypic patterns in the assays described in **a**. The methods in **a** are described in the supplementary text.
