# Supplementary material for: Flagella-related gene mutations in Vibrio cholerae during extended cultivation in nutrient-limited media impair cell motility and prolong culturability
Source: mSystems. 2023 Aug 29;8(5):e00109-23. doi: 10.1128/msystems.00109-23 (PMC10654082; doi:10.1128/msystems.00109-23)
Supplement: Table S3 — Mutation profiles of the 68 isolates derived from V. cholerae O1 MS84A. [file msystems.00109-23-s0009.pdf]

Table S3. Mutation profiles of the 68 isolates derived from *V. cholerae* O1 MS84A

no	Exp.	Isolate	Genotype	Incubation	Chromosome	Position	Mutation	Annotation	Gene	Function/Description	K-number
1	1	VC1	wild-type	day 60	I	283,156	G→T	E17* (GAA→TAA)	1335	AAA family ATPase	K03112
2	1	VC1	wild-type	day 60	I	328,764	A→G	T108A (ACC→GCC)	rpsM	30S ribosomal protein S13	K02952
3	1	VC1	wild-type	day 60	I	744,429	$(G)_{5\rightarrow 6}$	intergenic (+175/-5)	-	-	-
4	1	VC1	wild-type	day 60	I	796,837	T→C	R476R (CGT→CGC)	fliF	flagellar M-ring protein FliF	K02409
5	1	VC1	wild-type	day 60	I	1,278,054	A→G	E18G (GAG→GGG)	5930	DNA-3-methyladenine glycosylase I	K01246
6	1	VC1	wild-type	day 60	I	1,436,807	G→A	G127S (GGC→AGC)	nagK	N-acetylglucosamine kinase	K00884
7	1	VC1	wild-type	day 60	I	1,742,904	G→A	H418Y (CAC→TAC)	7865	imelysin family protein	K07231
8	1	VC1	wild-type	day 60	I	1,969,667	T→C	L159L (TTA→CTA)	8910	outer membrane protein transport protein	K06076
9	1	VC1	wild-type	day 60	I	2,187,993	$(C)_{9\rightarrow 8}$	coding (901/1470 nt)	tcpC	toxin-coregulated pilus secretin TcpC	K10932
10	1	VC1	wild-type	day 60	I	2,405,711	$(A)_{9\rightarrow 8}$	intergenic (-272/-284)	-	-	-
11	1	VC1	wild-type	day 60	I	2,515,306	G→A	G626S (GGT→AGT)	mutS	DNA mismatch repair protein MutS	K03555
12	1	VC1	wild-type	day 60	I	2,640,105	$(C)_{8\rightarrow 9}$	coding (1426/3759 nt)	11935	MSHA biogenesis protein MshQ	K12287
13	1	VC1	wild-type	day 60	I	2,810,365	$(T)_{9\rightarrow 8}$	coding (605/1200 nt)	manA	mannose-6-phosphate isomerase, class I	K01809
14	1	VC1	wild-type	day 60	I	2,893,874	G→A	D156N (GAC→AAC)	gorA	glutathione-disulfide reductase	K00383
15	1	VC1	wild-type	day 60	I	2,955,504	G→A	intergenic (-45/+430)	-	-	-
16	1	VC1	wild-type	day 60	I	2,960,595	C→T	M192I (ATG→ATA)	13480	amino acid ABC transporter ATP-binding protein	-
17	1	VC1	wild-type	day 60	II	819,853	A→G	V237A (GTT→GCT)	pyk	pyruvate kinase	K00873
18	1	VC1	wild-type	day 60	II	894,233	A→G	T95A (ACA→GCA)	17855	YeeE/YedE family protein	K07112
19	1	VC1	wild-type	day 60	II	980,668	T→C	L327P (CTG→CCG)	18255	OFA family MFS transporter	K08177
20	1	VC2	wild-type	day 60	I	746,739	G→A	G140D (GGT→GAT)	flgL	flagellar hook-associated protein FlgL	K02397
21	1	VC2	wild-type	day 60	I	1,086,976	Δ1 bp	coding (356/777 nt)	5065	TSUP family transporter	K07090
22	1	VC2	wild-type	day 60	I	1,463,601	G→T	L68M (CTG→ATG)	6745	hypothetical protein	not assigned
23	1	VC2	wild-type	day 60	I	2,961,709	Δ74 bp	coding (57-130/672 nt)	13485	amino acid ABC transporter permease	K16962
24	1	VC3	wild-type	day 60	I	857,878	G→A	G196D (GGT→GAT)	flhA	flagellar biosynthesis protein FlhA	K02400
25	1	VC3	wild-type	day 60	I	1,463,601	G→T	L68M (CTG→ATG)	6745	hypothetical protein	not assigned
26	1	VC3	wild-type	day 60	I	2,961,709	Δ74 bp	coding (57-130/672 nt)	13485	amino acid ABC transporter permease	K16962
27	1	VC4	wild-type	day 60	I	283,599	$(AACATAAG)_{1\rightarrow 2}$	coding (492/1476 nt)	1335	AAA family ATPase	K03112
28	1	VC4	wild-type	day 60	I	1,463,601	G→T	L68M (CTG→ATG)	6745	hypothetical protein	not assigned
29	1	VC4	wild-type	day 60	I	2,961,709	Δ74 bp	coding (57-130/672 nt)	13485	amino acid ABC transporter permease	K16962
30	1	VC31	wild-type	day 60	I	283,156	G→T	E17* (GAA→TAA)	1335	AAA family ATPase	K03112
31	1	VC31	wild-type	day 60	I	1,436,807	G→A	G127S (GGC→AGC)	nagK	N-acetylglucosamine kinase	K00884
32	1	VC31	wild-type	day 60	I	1,969,667	T→C	L159L (TTA→CTA)	8910	outer membrane protein transport protein	K06076
33	1	VC31	wild-type	day 60	I	2,515,306	G→A	G626S (GGT→AGT)	mutS	DNA mismatch repair protein MutS	K03555
34	1	VC31	wild-type	day 60	I	2,640,105	$(C)_{8\rightarrow 9}$	coding (1426/3759 nt)	11935	MSHA biogenesis protein MshQ	K12287
35	1	VC31	wild-type	day 60	I	2,810,365	$(T)_{9\rightarrow 8}$	coding (605/1200 nt)	manA	mannose-6-phosphate isomerase, class I	K01809
36	1	VC31	wild-type	day 60	I	2,893,874	G→A	D156N (GAC→AAC)	gorA	glutathione-disulfide reductase	K00383
37	1	VC31	wild-type	day 60	I	2,955,504	G→A	intergenic (-45/+430)	-	-	-
38	1	VC31	wild-type	day 60	I	2,960,595	C→T	M192I (ATG→ATA)	13480	amino acid ABC transporter ATP-binding protein	-
39	1	VC31	wild-type	day 60	II	819,853	A→G	V237A (GTT→GCT)	pyk	pyruvate kinase	K00873
40	1	VC32	wild-type	day 60	I	795,908	C→T	Q167* (CAA→TAA)	fliF	flagellar M-ring protein FliF	K02409
41	1	VC32	wild-type	day 60	I	1,463,601	G→T	L68M (CTG→ATG)	6745	hypothetical protein	not assigned

42	1	VC32	wild-type	day 60	I	2,961,709	Δ74 bp	coding (57-130/672 nt)	13485	amino acid ABC transporter permease	K16962
43	2	VC5	wild-type	day 60	I	792,321	$(G)_{7\rightarrow6}$	coding (1384/1467 nt)	flrA	sigma-54 dependent transcriptional regulator	K10941
44	2	VC5	wild-type	day 60	I	1,913,972	+TCT	coding (907/1197 nt)	ackA	acetate kinase	K00925
45	2	VC5	wild-type	day 60	I	2,390,213	C→A	G464C (GGT→TGT)	pnp	polyribonucleotide nucleotidyltransferase	K00962
46	2	VC6	wild-type	day 60	I	792,321	$(G)_{7\rightarrow6}$	coding (1384/1467 nt)	flrA	sigma-54 dependent transcriptional regulator	K10941
47	2	VC6	wild-type	day 60	I	1,913,972	+TCT	coding (907/1197 nt)	ackA	acetate kinase	K00925
48	2	VC6	wild-type	day 60	I	2,390,213	C→A	G464C (GGT→TGT)	pnp	polyribonucleotide nucleotidyltransferase	K00962
49	2	VC7	wild-type	day 60	I	792,321	$(G)_{7\rightarrow8}$	coding (1384/1467 nt)	flrA	sigma-54 dependent transcriptional regulator	K10941
50	2	VC7	wild-type	day 60	I	1,216,051	$(A)_{5\rightarrow4}$	coding (2183/2631 nt)	topA	type I DNA topoisomerase	K03168
51	2	VC7	wild-type	day 60	I	1,785,517	C→T	A18T (GCA→ACA)	pheS	phenylalanine--tRNA ligase subunit alpha	K01889
52	2	VC7	wild-type	day 60	I	1,875,001	C→T	A238T (GCA→ACA)	hisA	histidine biosynthesis	K01814
53	2	VC7	wild-type	day 60	I	1,913,097	G→A	G11D (GGT→GAT)	ackA	acetate kinase	K00925
54	2	VC7	wild-type	day 60	I	2,339,950	C→T	P466S (CCT→TCT)	10540	sensor histidine kinase	K02478
55	2	VC7	wild-type	day 60	I	2,719,808	$(T)_{6\rightarrow5}$	coding (323/1719 nt)	12325	N-acetylmuramoyl-L-alanine amidase	K01448
56	2	VC8	wild-type	day 60	I	116,216	C→A	I228I (ATC→ATA)	yigB	5-amino-6-(5-phospho-D-ribitylamino)uracil phosphatase	K20862
57	2	VC8	wild-type	day 60	I	792,321	$(G)_{7\rightarrow6}$	coding (1384/1467 nt)	flrA	sigma-54 dependent transcriptional regulator	K10941
58	2	VC8	wild-type	day 60	I	1,913,972	+TCT	coding (907/1197 nt)	ackA	acetate kinase	K00925
59	2	VC8	wild-type	day 60	I	2,390,213	C→A	G464C (GGT→TGT)	pnp	polyribonucleotide nucleotidyltransferase	K00962
60	2	VC8	wild-type	day 60	II	698,806	C→T	P185L (CCG→CTG)	phhA	phenylalanine 4-monooxygenase	K00500
61	2	VC33	wild-type	day 60	I	116,216	C→A	I228I (ATC→ATA)	yigB	5-amino-6-(5-phospho-D-ribitylamino)uracil phosphatase	K20862
62	2	VC33	wild-type	day 60	I	792,321	$(G)_{7\rightarrow6}$	coding (1384/1467 nt)	flrA	sigma-54 dependent transcriptional regulator	K10941
63	2	VC33	wild-type	day 60	I	1,913,972	+TCT	coding (907/1197 nt)	ackA	acetate kinase	K00925
64	2	VC33	wild-type	day 60	I	2,390,213	C→A	G464C (GGT→TGT)	pnp	polyribonucleotide nucleotidyltransferase	K00962
65	2	VC33	wild-type	day 60	II	698,806	C→T	P185L (CCG→CTG)	phhA	phenylalanine 4-monooxygenase	K00500
66	2	VC34	wild-type	day 60	I	116,216	C→A	I228I (ATC→ATA)	yigB	5-amino-6-(5-phospho-D-ribitylamino)uracil phosphatase	K20862
67	2	VC34	wild-type	day 60	I	792,321	$(G)_{7\rightarrow6}$	coding (1384/1467 nt)	flrA	sigma-54 dependent transcriptional regulator	K10941
68	2	VC34	wild-type	day 60	I	1,913,972	+TCT	coding (907/1197 nt)	ackA	acetate kinase	K00925
69	2	VC34	wild-type	day 60	I	2,390,213	C→A	G464C (GGT→TGT)	pnp	polyribonucleotide nucleotidyltransferase	K00962
70	2	VC34	wild-type	day 60	II	698,806	C→T	P185L (CCG→CTG)	phhA	phenylalanine 4-monooxygenase	K00500
71	2	VC35	wild-type	day 60	I	792,321	$(G)_{7\rightarrow6}$	coding (1384/1467 nt)	flrA	sigma-54 dependent transcriptional regulator	K10941
72	2	VC35	wild-type	day 60	I	1,913,972	+TCT	coding (907/1197 nt)	ackA	acetate kinase	K00925
73	2	VC35	wild-type	day 60	I	2,390,213	C→A	G464C (GGT→TGT)	pnp	polyribonucleotide nucleotidyltransferase	K00962
74	2	VC35	wild-type	day 60	I	2,819,554	$(A)_{5\rightarrow4}$	coding (1316/1866 nt)	12800	polysaccharide biosynthesis protein	K24300
75	2	VC36	wild-type	day 60	I	792,321	$(G)_{7\rightarrow6}$	coding (1384/1467 nt)	flrA	sigma-54 dependent transcriptional regulator	K10941
76	2	VC36	wild-type	day 60	I	1,913,972	+TCT	coding (907/1197 nt)	ackA	acetate kinase	K00925
77	2	VC36	wild-type	day 60	I	2,390,213	C→A	G464C (GGT→TGT)	pnp	polyribonucleotide nucleotidyltransferase	K00962
78	2	VC36	wild-type	day 60	I	2,819,554	$(A)_{5\rightarrow4}$	coding (1316/1866 nt)	12800	polysaccharide biosynthesis protein	K24300
79	2	VC37	wild-type	day 60	I	792,321	$(G)_{7\rightarrow6}$	coding (1384/1467 nt)	flrA	sigma-54 dependent transcriptional regulator	K10941
80	2	VC37	wild-type	day 60	I	1,913,972	+TCT	coding (907/1197 nt)	ackA	acetate kinase	K00925
81	2	VC37	wild-type	day 60	I	2,390,213	C→A	G464C (GGT→TGT)	pnp	polyribonucleotide nucleotidyltransferase	K00962
82	2	VC37	wild-type	day 60	I	2,819,554	$(A)_{5\rightarrow4}$	coding (1316/1866 nt)	12800	polysaccharide biosynthesis protein	K24300
83	3	VC13	wild-type	day 60	I	1,913,585	C→T	R174C (CGC→TGC)	ackA	acetate kinase	K00925
84	3	VC13	wild-type	day 60	I	2,779,649	G→A	W414* (TGG→TGA)	acs	acetate--CoA ligase	K01895
85	3	VC13	wild-type	day 60	I	2,915,821	G→A	intergenic (-135/+157)	-	-	-
86	3	VC14	wild-type	day 60	I	171,065	Δ2 bp	coding (1317-1318/1629 nt)	pckA	phosphoenolpyruvate carboxykinase (ATP)	K01610

87	3	VC14	wild-type	day 60	I	1,913,585	C→T	R174C (CGC→TGC)	ackA	acetate kinase	K00925
88	3	VC14	wild-type	day 60	I	2,915,821	G→A	intergenic (-135/+157)	-	-	-
89	3	VC14	wild-type	day 60	II	862,985	Δ5 bp	coding (697-701/1011 nt)	17720	LacI family DNA-binding transcriptional regulator	not assigned
90	3	VC15	wild-type	day 60	I	792,321	$(G)_{7\to8}$	coding (1384/1467 nt)	flrA	sigma-54 dependent transcriptional regulator	K10941
91	3	VC15	wild-type	day 60	I	1,025,376	T→G	Y197D (TAT→GAT)	cysB	HTH-type transcriptional regulator CysB	K13634
92	3	VC15	wild-type	day 60	I	1,090,287	A→G	D111G (GAC→GGC)	ompT	porin OmpT	K10940
93	3	VC15	wild-type	day 60	I	1,913,135	Δ1 bp	coding (70/1197 nt)	ackA	acetate kinase	K00925
94	3	VC15	wild-type	day 60	I	2,658,837	Δ84 bp	coding (584-667/873 nt)	galU	UTP--glucose-1-phosphate uridylyltransferase GalU	K00963
95	3	VC16	wild-type	day 60	I	792,321	$(G)_{7\to8}$	coding (1384/1467 nt)	flrA	sigma-54 dependent transcriptional regulator	K10941
96	3	VC16	wild-type	day 60	I	1,025,376	T→G	Y197D (TAT→GAT)	cysB	HTH-type transcriptional regulator CysB	K13634
97	3	VC16	wild-type	day 60	I	1,090,287	A→G	D111G (GAC→GGC)	ompT	porin OmpT	K10940
98	3	VC16	wild-type	day 60	I	1,117,854	C→A	L149F (TTG→TTT)	5215	PTS sugar transporter subunit IIA	K02806
99	3	VC16	wild-type	day 60	I	1,749,856	G→A	T649I (ACC→ATC)	gyrA	DNA topoisomerase (ATP-hydrolyzing) subunit A	K02469
100	3	VC16	wild-type	day 60	I	2,658,837	Δ84 bp	coding (584-667/873 nt)	galU	UTP--glucose-1-phosphate uridylyltransferase GalU	K00963
101	3	VC41	wild-type	day 60	I	791,081	G→A	W48* (TGG→TGA)	flrA	sigma-54 dependent transcriptional regulator	K10941
102	3	VC41	wild-type	day 60	I	1,913,331	G→A	R89H (CGC→CAC)	ackA	acetate kinase	K00925
103	3	VC41	wild-type	day 60	I	2,339,623	C→A	Q357K (CAG→AAG)	10540	sensor histidine kinase	K02478
104	3	VC41	wild-type	day 60	II	698,941	Δ18 bp	coding (689-706/795 nt)	phhA	phenylalanine 4-monooxygenase	K00500
105	3	VC42	wild-type	day 60	I	791,081	G→A	W48* (TGG→TGA)	flrA	sigma-54 dependent transcriptional regulator	K10941
106	3	VC42	wild-type	day 60	I	1,913,331	G→A	R89H (CGC→CAC)	ackA	acetate kinase	K00925
107	3	VC42	wild-type	day 60	I	2,339,623	C→A	Q357K (CAG→AAG)	10540	sensor histidine kinase	K02478
108	3	VC42	wild-type	day 60	II	698,941	Δ18 bp	coding (689-706/795 nt)	phhA	phenylalanine 4-monooxygenase	K00500
109	3	VC43	wild-type	day 60	I	171,520	G→A	A288V (GCG→GTG)	pckA	phosphoenolpyruvate carboxykinase (ATP)	K01610
110	3	VC43	wild-type	day 60	I	835,807	C→T	H518Y (CAC→TAC)	sucA	2-oxoglutarate dehydrogenase E1 component	K00164
111	3	VC43	wild-type	day 60	I	1,866,895	Δ1 bp	coding (2086/2271 nt)	clpA	ATP-dependent Clp protease ATP-binding subunit ClpA	K03694
112	3	VC43	wild-type	day 60	I	2,390,641	$(T)_{5\to4}$	coding (962/2130 nt)	pnp	polyribonucleotide nucleotidyltransferase	K00962
113	3	VC43	wild-type	day 60	I	2,719,742	$(TCAATGA)_{1\to2}$	coding (389/1719 nt)	12325	N-acetylmuramoyl-L-alanine amidase	K01448
114	4	VC9	wild-type	day 60	I	395,172	$(TCTTCAC)_{1\to2}$	coding (668/1512 nt)	pepA	leucyl aminopeptidase	K01255
115	4	VC9	wild-type	day 60	I	791,162	$(ATATC)_{1\to2}$	coding (225/1467 nt)	flrA	sigma-54 dependent transcriptional regulator	K10941
116	4	VC9	wild-type	day 60	I	1,215,353	$(TTAGAAAAG)_{1\to2}$	coding (1485/2631 nt)	topA	type I DNA topoisomerase	K03168
117	4	VC9	wild-type	day 60	I	1,913,616	+TT	coding (551/1197 nt)	ackA	acetate kinase	K00925
118	4	VC9	wild-type	day 60	I	2,307,677	$(AGCGCGTGTTTCAC)_{1\to2}$	coding (1192/1302 nt)	phoR	phosphate regulon sensor histidine kinase PhoR	K07636
119	4	VC10	wild-type	day 60	I	292,831	$(AATAA)_{1\to2}$	coding (596/1413 nt)	rimO	30S ribosomal protein S12 methylthiotransferase RimO	K14441
120	4	VC10	wild-type	day 60	I	394,487	$(GAAAA)_{1\to2}$	coding (1353/1512 nt)	pepA	leucyl aminopeptidase	K01255
121	4	VC10	wild-type	day 60	I	791,162	$(ATATC)_{1\to2}$	coding (225/1467 nt)	flrA	sigma-54 dependent transcriptional regulator	K10941
122	4	VC10	wild-type	day 60	I	1,215,353	$(TTAGAAAAG)_{1\to2}$	coding (1485/2631 nt)	topA	type I DNA topoisomerase	K03168
123	4	VC10	wild-type	day 60	I	1,913,616	+TT	coding (551/1197 nt)	ackA	acetate kinase	K00925
124	4	VC10	wild-type	day 60	I	2,050,346	Δ3 bp	coding (208-210/258 nt)	9260	HPr family phosphocarrier protein	K02784
125	4	VC10	wild-type	day 60	I	2,526,759	Δ8 bp	intergenic (-71/-163)	-	-	-
126	4	VC10	wild-type	day 60	I	2,834,991	$(TTAATA)_{1\to2}$	coding (1298/1392 nt)	12875	phosphomannomutase CpsG	K01840
127	4	VC10	wild-type	day 60	II	267,987	+TC	coding (523/657 nt)	tagO	type VI secretion system-associated protein TagO	K11909
128	4	VC11	wild-type	day 60	I	395,389	G→A	R151C (CGC→TGC)	pepA	leucyl aminopeptidase	K01255
129	4	VC11	wild-type	day 60	I	594,447	C→T	L875L (CTG→TTG)	2750	beta-galactosidase	K01190
130	4	VC11	wild-type	day 60	I	2,339,579	G→T	G342V (GGC→GTC)	10540	sensor histidine kinase	K02478
131	4	VC11	wild-type	day 60	I	2,458,321	C→T	A73V (GCT→GTT)	dksA	RNA polymerase-binding protein DksA	K06204

132	4	VC11	wild-type	day 60	I	2,836,054	C→A	V79F (GTT→TTT)	12875	phosphomannomutase CpsG	K01840
133	4	VC12	wild-type	day 60	I	791,162	$(ATATC)_{1\to2}$	coding (225/1467 nt)	flrA	sigma-54 dependent transcriptional regulator	K10941
134	4	VC12	wild-type	day 60	I	1,215,353	$(TTAGAAAAG)_{1\to2}$	coding (1485/2631 nt)	topA	type I DNA topoisomerase	K03168
135	4	VC12	wild-type	day 60	I	1,913,616	+TT	coding (551/1197 nt)	ackA	acetate kinase	K00925
136	4	VC12	wild-type	day 60	I	2,083,172	+ATTA	intergenic (-403/-87)	-	-	-
137	4	VC12	wild-type	day 60	II	698,239	Δ1 bp	intergenic (-220/-14)	-	-	-
138	4	VC38	wild-type	day 60	I	395,172	$(TCTTCAC)_{1\to2}$	coding (668/1512 nt)	pepA	leucyl aminopeptidase	K01255
139	4	VC38	wild-type	day 60	I	791,162	$(ATATC)_{1\to2}$	coding (225/1467 nt)	flrA	sigma-54 dependent transcriptional regulator	K10941
140	4	VC38	wild-type	day 60	I	1,078,976	Δ2 bp	intergenic (-405/-311)	-	-	-
141	4	VC38	wild-type	day 60	I	1,215,353	$(TTAGAAAAG)_{1\to2}$	coding (1485/2631 nt)	topA	type I DNA topoisomerase	K03168
142	4	VC38	wild-type	day 60	I	1,801,985	Δ1 bp	coding (542/1011 nt)	hutG	formimidoylglutamase	K01479
143	4	VC38	wild-type	day 60	I	1,913,616	+TT	coding (551/1197 nt)	ackA	acetate kinase	K00925
144	4	VC38	wild-type	day 60	II	994,251	Δ1 bp	coding (503/1452 nt)	focA	formate transporter FocA	K06212
145	4	VC39	wild-type	day 60	I	395,715	+T	coding (125/1512 nt)	pepA	leucyl aminopeptidase	K01255
146	4	VC39	wild-type	day 60	I	791,834	Δ4 bp	coding (897-900/1467 nt)	flrA	sigma-54 dependent transcriptional regulator	K10941
147	4	VC39	wild-type	day 60	I	1,215,353	$(TTAGAAAAG)_{1\to2}$	coding (1485/2631 nt)	topA	type I DNA topoisomerase	K03168
148	4	VC39	wild-type	day 60	I	1,913,616	+TT	coding (551/1197 nt)	ackA	acetate kinase	K00925
149	4	VC39	wild-type	day 60	I	2,835,827	+T	coding (462/1392 nt)	12875	phosphomannomutase CpsG	K01840
150	4	VC40	wild-type	day 60	I	227,575	C→T	V213I (GTT→ATT)	metF	methylenetetrahydrofolate reductase	K00297
151	4	VC40	wild-type	day 60	I	1,391,620	C→T	A234V (GCG→GTG)	6390	LysR family transcriptional regulator	K03717
152	4	VC40	wild-type	day 60	I	1,913,526	Δ1 bp	coding (461/1197 nt)	ackA	acetate kinase	K00925
153	5	D10MT3	wild-type	day10	-	-	-	-	-	No mutation found	-
154	5	D10MT2	wild-type	day10	I	2,556,900	Δ1 bp	intergenic (+289/-473)	-	-	-
155	5	D10MT4	wild-type	day10	I	2,836,400	C→T	G430D (GGT→GAT)	manC	mannose-1-phosphate guanylyltransferase	K00971
156	5	D10NM8	wild-type	day10	I	793,990	Δ39 bp	coding (417-455/1434 nt)	flrC	sigma-54 dependent transcriptional regulator	K10943
157	5	D20MT5	wild-type	day 20	I	2,832,218	Δ7 bp	coding (430-436/1383 nt)	12860	glycosyltransferase	-
158	5	D20MT6	wild-type	day 20	I	869,595	(ATTTG)2→3	coding (41/504 nt)	flrD	flagellar transcriptional regulator FlrD	-
159	5	D20PM3	wild-type	day 20	I	799,908	Δ2 bp	coding (821-822/1320 nt)	fliI	flagellar protein export ATPase FliI	K02412
160	5	D20NM9	wild-type	day 20	I	739,869	Δ9 bp	coding (98-106/750 nt)	flgF	flagellar basal-body rod protein FlgF	K02391
161	5	D30MT7	wild-type	day 30	I	1,923,669	C→G	intergenic (-328/-296)	-	-	-
162	5	D30MT8	wild-type	day 30	I	857,772	C→T	P161S (CCC→TCC)	flhA	flagellar biosynthesis protein FlhA	K02400
163	5	D30PM4	wild-type	day 30	I	2,720,097	Δ1 bp	coding (34/1719 nt)	12325	N-acetylmuramoyl-L-alanine amidase	K01448
164	5	D30PM4	wild-type	day 30	I	792,321	$(G)_{7\to8}$	coding (1384/1467 nt)	flrA	sigma-54 dependent transcriptional regulator	K10941
165	5	D30NM10	wild-type	day 30	I	2,819,677	T→C	I480T (ATA→ACA)	12800	polysaccharide biosynthesis protein	K24300
166	5	D30NM10	wild-type	day 30	I	797,613	C→T	Q157* (CAA→TAA)	fliG	flagellar motor switch protein FliG	K02410
167	5	D60MT15	wild-type	day 60	I	1,463,543	C→T	S87N (AGC→AAC)	6745	hypothetical protein	-
168	5	D60MT16	wild-type	day 60	I	1,913,392	Δ1 bp	coding (327/1197 nt)	ackA	acetate kinase	K00925
169	5	D60MT16	wild-type	day 60	I	788,731	G→T	E252* (GAA→TAA)	fliD	flagellar filament capping protein FliD	K02407
170	5	D60PM8	wild-type	day 60	I	2,810,365	(T)9→8	coding (605/1200 nt)	manA	mannose-6-phosphate isomerase, class I	K01809
171	5	D60PM8	wild-type	day 60	II	290,241	(A)6→5	coding (1209/1557 nt)	15255	EAL domain-containing protein	K21090
172	5	D60PM8	wild-type	day 60	II	70,472	A→T	V6E (GTG→GAG)	14210	acetyltransferase	-
173	5	D60PM8	wild-type	day 60	I	2,720,097	Δ1 bp	coding (34/1719 nt)	12325	N-acetylmuramoyl-L-alanine amidase	K01448
174	5	D60PM8	wild-type	day 60	I	1,913,328	Δ3 bp	coding (263-265/1197 nt)	ackA	acetate kinase	K00925
175	5	D60PM8	wild-type	day 60	I	1,847,355	(C)11→10	coding (242/738 nt)	8340	ATP-dependent zinc protease	-
176	5	D60PM8	wild-type	day 60	I	792,321	$(G)_{7\to8}$	coding (1384/1467 nt)	flrA	sigma-54 dependent transcriptional regulator	K10941

177	5	D60NM14	wild-type	day 60	I	2,719,345	Δ141 bp	coding (646-786/1719 nt)	12325	N-acetylmuramoyl-L-alanine amidase	K01448
178	5	D60NM14	wild-type	day 60	I	2,339,786	G→A	S411N (AGC→AAC)	10540	sensor histidine kinase	K02478
179	5	D60NM14	wild-type	day 60	I	1,913,140	Δ320 bp	coding (75-394/1197 nt)	ackA	acetate kinase	K00925
180	5	D60NM14	wild-type	day 60	I	791,140	Δ1 bp	coding (203/1467 nt)	flrA	sigma-54 dependent transcriptional regulator	K10941
181	6	D10MT9	wild-type	day10	-	-	-	-	-	No mutation found	-
182	6	D10MT10	wild-type	day10	I	2,820,389	C→T	P93L (CCA→CTA)	12805	glycosyltransferase family 4 protein	-
183	6	D10PM5	wild-type	day10	I	860,274	Δ1 bp	coding (855/1488 nt)	flhF	flagellar biosynthesis protein FlhF	K02404
184	6	D10PM5	wild-type	day10	II	211,132	(ACAGCA)19→20	coding (1442/1917 nt)	14945	VWA domain-containing protein	K07114
185	6	D10NM11	wild-type	day10	I	856,120	Δ1,229 bp	Deletion	3 genes		
186	6	D10NM11	wild-type	day10	I	856,120	Δ1,229 bp	Deletion	flhA	flagellar biosynthesis protein FlhA	K02400
187	6	D10NM11	wild-type	day10	I	856,120	Δ1,229 bp	Deletion	4040	insulinase family protein	-
188	6	D10NM11	wild-type	day10	I	856,120	Δ1,229 bp	Deletion	sixA	phosphohistidine phosphatase SixA	K08296
189	6	D20MT11	wild-type	day 20	II	211,132	(ACAGCA)19→20	coding (1442/1917 nt)	14945	VWA domain-containing protein	K07114
190	6	D20MT11	wild-type	day 20	I	1,759,302	C→T	A20V (GCA→GTA)	7930	DUF3943 domain-containing protein	-
191	6	D20MT12	wild-type	day 20	II	211,132	(ACAGCA)19→20	coding (1442/1917 nt)	14945	VWA domain-containing protein	K07114
192	6	D20MT12	wild-type	day 20	I	1,759,302	C→T	A20V (GCA→GTA)	7930	DUF3943 domain-containing protein	-
193	6	D20PM6	wild-type	day 20	II	211,132	(ACAGCA)19→20	coding (1442/1917 nt)	14945	VWA domain-containing protein	K07114
194	6	D20PM6	wild-type	day 20	I	805,849	C→T	T117I (ACC→ATC)	fliP	flagellar type III secretion system pore protein FliP	K02419
195	6	D20PM6	wild-type	day 20	I	1,510,978	Δ6,909 bp	Deletion	9 genes		
196	6	D20PM6	wild-type	day 20	I	1,510,978	Δ6,909 bp	Deletion	6950	helix-turn-helix transcriptional regulator	-
197	6	D20PM6	wild-type	day 20	I	1,510,978	Δ6,909 bp	Deletion	6955	replication initiation factor domain-containing protein	-
198	6	D20PM6	wild-type	day 20	I	1,510,978	Δ6,909 bp	Deletion	6960	hypothetical protein	-
199	6	D20PM6	wild-type	day 20	I	1,510,978	Δ6,909 bp	Deletion	6965	colonization factor	-
200	6	D20PM6	wild-type	day 20	I	1,510,978	Δ6,909 bp	Deletion	6970	minor coat protein pIII	-
201	6	D20PM6	wild-type	day 20	I	1,510,978	Δ6,909 bp	Deletion	ace	accessory cholera enterotoxin	-
202	6	D20PM6	wild-type	day 20	I	1,510,978	Δ6,909 bp	Deletion	zot	zonula occludens toxin ZOT	K10954
203	6	D20PM6	wild-type	day 20	I	1,510,978	Δ6,909 bp	Deletion	ctxA	cholera enterotoxin catalytic subunit CtxA	K10928
204	6	D20PM6	wild-type	day 20	I	1,510,978	Δ6,909 bp	Deletion	ctxB	cholera enterotoxin binding subunit CtxB	K10929
205	6	D20PM6	wild-type	day 20	I	2,215,028	(A)8→7	intergenic (+57/-13)	-	-	-
206	6	D20NM12	wild-type	day 20	II	211,132	(ACAGCA)19→20	coding (1442/1917 nt)	14945	VWA domain-containing protein	K07114
207	6	D20NM12	wild-type	day 20	I	744,687	+CTTT	coding (254/1875 nt)	flgK	flagellar hook-associated protein FlgK	K02396
208	6	D30MT13	wild-type	day 30	II	211,132	(ACAGCA)19→20	coding (1442/1917 nt)	14945	VWA domain-containing protein	K07114
209	6	D30MT13	wild-type	day 30	I	2,824,972	+AGA	coding (1954/2478 nt)	12830	acyl-CoA reductase	-
210	6	D30MT13	wild-type	day 30	I	1,759,302	C→T	A20V (GCA→GTA)	7930	DUF3943 domain-containing protein	-
211	6	D30MT13	wild-type	day 30	I	2,491,870	Δ1 bp	intergenic (-103/+9)	-	-	-
212	6	D30MT14	wild-type	day 30	II	211,132	(ACAGCA)19→20	coding (1442/1917 nt)	14945	VWA domain-containing protein	K07114
213	6	D30MT14	wild-type	day 30	I	1,759,302	C→T	A20V (GCA→GTA)	7930	DUF3943 domain-containing protein	-
214	6	D30PM7	wild-type	day 30	I	791,401	C→A	S155* (TCG→TAG)	flrA	sigma-54 dependent transcriptional regulator	K10941
215	6	D30NM13	wild-type	day 30	I	2,390,242	G→A	A454V (GCC→GTC)	pnp	polyribonucleotide nucleotidyltransferase	K00962
216	6	D30NM13	wild-type	day 30	I	795,451	Δ3,315 bp	Deletion	3 genes		
217	6	D30NM13	wild-type	day 30	I	795,451	Δ3,315 bp	Deletion	fliH	flagellar assembly protein FliH	K02411
218	6	D30NM13	wild-type	day 30	I	795,451	Δ3,315 bp	Deletion	fliG	flagellar motor switch protein FliG	K02410
219	6	D30NM13	wild-type	day 30	I	795,451	Δ3,315 bp	Deletion	fliF	flagellar M-ring protein FliF	K02409
220	6	D60MT17	wild-type	day 60	I	1,759,302	C→T	A20V (GCA→GTA)	7930	DUF3943 domain-containing protein	-
221	6	D60MT17	wild-type	day 60	I	1,923,669	C→G	intergenic (-328/-296)	-	-	-

222	6	D60MT17	wild-type	day 60	II	211,132	(ACAGCA)19→20	coding (1442/1917 nt)	14945	VWA domain-containing protein	K07114
223	6	D60MT17	wild-type	day 60	II	690,259	Δ19,862 bp	Deletion	20 genes		
224	6	D60MT17	wild-type	day 60	II	690,259	Δ19,862 bp	Deletion	16970	sugar O-acetyltransferase	K00661
225	6	D60MT17	wild-type	day 60	II	690,259	Δ19,862 bp	Deletion	16975	aromatic amino acid DMT transporter YddG	-
226	6	D60MT17	wild-type	day 60	II	690,259	Δ19,862 bp	Deletion	16980	cation diffusion facilitator family transporter	-
227	6	D60MT17	wild-type	day 60	II	690,259	Δ19,862 bp	Deletion	16985	LysR family transcriptional regulator	-
228	6	D60MT17	wild-type	day 60	II	690,259	Δ19,862 bp	Deletion	16990	methyltransferase domain-containing protein	-
229	6	D60MT17	wild-type	day 60	II	690,259	Δ19,862 bp	Deletion	16995	hypothetical protein	-
230	6	D60MT17	wild-type	day 60	II	690,259	Δ19,862 bp	Deletion	17000	LysR family transcriptional regulator	-
231	6	D60MT17	wild-type	day 60	II	690,259	Δ19,862 bp	Deletion	17005	acetoacetate--CoA ligase	K01907
232	6	D60MT17	wild-type	day 60	II	690,259	Δ19,862 bp	Deletion	phhA	phenylalanine 4-monooxygenase	K00500
233	6	D60MT17	wild-type	day 60	II	690,259	Δ19,862 bp	Deletion	17015	4a-hydroxytetrahydrobiopterin dehydratase	K01724
234	6	D60MT17	wild-type	day 60	II	690,259	Δ19,862 bp	Deletion	17020	diaminobutyrate acetyltransferase	K06718
235	6	D60MT17	wild-type	day 60	II	690,259	Δ19,862 bp	Deletion	17025	diaminobutyrate--2-oxoglutarate transaminase	K00836
236	6	D60MT17	wild-type	day 60	II	690,259	Δ19,862 bp	Deletion	17030	ectoine synthase	K06720
237	6	D60MT17	wild-type	day 60	II	690,259	Δ19,862 bp	Deletion	17035	aspartate kinase	K00928
238	6	D60MT17	wild-type	day 60	II	690,259	Δ19,862 bp	Deletion	17040	hypothetical protein	-
239	6	D60MT17	wild-type	day 60	II	690,259	Δ19,862 bp	Deletion	17045	chaperonin GroEL	K04077
240	6	D60MT17	wild-type	day 60	II	690,259	Δ19,862 bp	Deletion	17050	co-chaperone GroES	K04078
241	6	D60MT17	wild-type	day 60	II	690,259	Δ19,862 bp	Deletion	17055	magnesium transporter	K06213
242	6	D60MT17	wild-type	day 60	II	690,259	Δ19,862 bp	Deletion	17060	mechanosensitive ion channel family protein	-
243	6	D60MT17	wild-type	day 60	II	690,259	Δ19,862 bp	Deletion	speA	arginine decarboxylase	K01585
244	6	D60MT18	wild-type	day 60	I	1,759,302	C→T	A20V (GCA→GTA)	7930	DUF3943 domain-containing protein	-
245	6	D60MT18	wild-type	day 60	I	1,923,669	C→G	intergenic (-328/-296)	-	-	-
246	6	D60MT18	wild-type	day 60	II	690,259	Δ19,862 bp	Deletion	20 genes		
247	6	D60MT18	wild-type	day 60	II	690,259	Δ19,862 bp	Deletion	16970	sugar O-acetyltransferase	K00661
248	6	D60MT18	wild-type	day 60	II	690,259	Δ19,862 bp	Deletion	16975	aromatic amino acid DMT transporter YddG	-
249	6	D60MT18	wild-type	day 60	II	690,259	Δ19,862 bp	Deletion	16980	cation diffusion facilitator family transporter	-
250	6	D60MT18	wild-type	day 60	II	690,259	Δ19,862 bp	Deletion	16985	LysR family transcriptional regulator	-
251	6	D60MT18	wild-type	day 60	II	690,259	Δ19,862 bp	Deletion	16990	methyltransferase domain-containing protein	-
252	6	D60MT18	wild-type	day 60	II	690,259	Δ19,862 bp	Deletion	16995	hypothetical protein	-
253	6	D60MT18	wild-type	day 60	II	690,259	Δ19,862 bp	Deletion	17000	LysR family transcriptional regulator	-
254	6	D60MT18	wild-type	day 60	II	690,259	Δ19,862 bp	Deletion	17005	acetoacetate--CoA ligase	K01907
255	6	D60MT18	wild-type	day 60	II	690,259	Δ19,862 bp	Deletion	phhA	phenylalanine 4-monooxygenase	K00500
256	6	D60MT18	wild-type	day 60	II	690,259	Δ19,862 bp	Deletion	17015	4a-hydroxytetrahydrobiopterin dehydratase	K01724
257	6	D60MT18	wild-type	day 60	II	690,259	Δ19,862 bp	Deletion	17020	diaminobutyrate acetyltransferase	K06718
258	6	D60MT18	wild-type	day 60	II	690,259	Δ19,862 bp	Deletion	17025	diaminobutyrate--2-oxoglutarate transaminase	K00836
259	6	D60MT18	wild-type	day 60	II	690,259	Δ19,862 bp	Deletion	17030	ectoine synthase	K06720
260	6	D60MT18	wild-type	day 60	II	690,259	Δ19,862 bp	Deletion	17035	aspartate kinase	K00928
261	6	D60MT18	wild-type	day 60	II	690,259	Δ19,862 bp	Deletion	17040	hypothetical protein	-
262	6	D60MT18	wild-type	day 60	II	690,259	Δ19,862 bp	Deletion	17045	chaperonin GroEL	K04077
263	6	D60MT18	wild-type	day 60	II	690,259	Δ19,862 bp	Deletion	17050	co-chaperone GroES	K04078
264	6	D60MT18	wild-type	day 60	II	690,259	Δ19,862 bp	Deletion	17055	magnesium transporter	K06213
265	6	D60MT18	wild-type	day 60	II	690,259	Δ19,862 bp	Deletion	17060	mechanosensitive ion channel family protein	-
266	6	D60MT18	wild-type	day 60	II	690,259	Δ19,862 bp	Deletion	speA	arginine decarboxylase	K01585

267	6	D60PM9	wild-type	day 60	I	1,090,287	A→G	D111G (GAC→GGC)	ompT	porin OmpT	K10940
268	6	D60PM9	wild-type	day 60	II	845,204	C→T	P357S (CCG→TCG)	17640	class I poly(R)-hydroxyalkanoic acid synthase	K03821
269	6	D60PM9	wild-type	day 60	II	211,132	(ACAGCA)19→20	coding (1442/1917 nt)	14945	VWA domain-containing protein	K07114
270	6	D60PM9	wild-type	day 60	I	2,820,749	C→A	A213E (GCA→GAA)	12805	glycosyltransferase family 4 protein	-
271	6	D60PM9	wild-type	day 60	I	1,911,025	Δ5,723 bp	Deletion	6 genes		
272	6	D60PM9	wild-type	day 60	I	1,911,025	Δ5,723 bp	Deletion	8660	glutathione S-transferase family protein	K07393
273	6	D60PM9	wild-type	day 60	I	1,911,025	Δ5,723 bp	Deletion	pta	phosphate acetyltransferase	K13788
274	6	D60PM9	wild-type	day 60	I	1,911,025	Δ5,723 bp	Deletion	ackA	acetate kinase	K00925
275	6	D60PM9	wild-type	day 60	I	1,911,025	Δ5,723 bp	Deletion	8645	DUF412 domain-containing protein	K09899
276	6	D60PM9	wild-type	day 60	I	1,911,025	Δ5,723 bp	Deletion	8640	VF530 family DNA-binding protein	-
277	6	D60PM9	wild-type	day 60	I	1,911,025	Δ5,723 bp	Deletion	8635	ABC transporter substrate-binding protein	K01989
278	6	D60PM9	wild-type	day 60	I	1,510,439	G→A	K55K (AAG→AAA)	6940	hypothetical protein	-
279	6	D60PM9	wild-type	day 60	I	791,679	A→T	K248* (AAA→TAA)	flrA	sigma-54 dependent transcriptional regulator	K10941
280	6	D60PM10	wild-type	day 60	I	2,390,242	G→A	A454V (GCC→GTC)	pnp	polyribonucleotide nucleotidyltransferase	K00962
281	6	D60PM10	wild-type	day 60	I	1,090,287	A→G	D111G (GAC→GGC)	ompT	porin OmpT	K10940
282	6	D60PM10	wild-type	day 60	I	1,921,799	G→A	R515C (CGT→TGT)	8685	peptide ABC transporter substrate-binding protein	K15580
283	6	D60PM10	wild-type	day 60	I	1,911,025	Δ5,723 bp	Deletion	6 genes		
284	6	D60PM10	wild-type	day 60	I	1,911,025	Δ5,723 bp	Deletion	8660	glutathione S-transferase family protein	K07393
285	6	D60PM10	wild-type	day 60	I	1,911,025	Δ5,723 bp	Deletion	pta	phosphate acetyltransferase	K13788
286	6	D60PM10	wild-type	day 60	I	1,911,025	Δ5,723 bp	Deletion	ackA	acetate kinase	K00925
287	6	D60PM10	wild-type	day 60	I	1,911,025	Δ5,723 bp	Deletion	8645	DUF412 domain-containing protein	K09899
288	6	D60PM10	wild-type	day 60	I	1,911,025	Δ5,723 bp	Deletion	8640	VF530 family DNA-binding protein	-
289	6	D60PM10	wild-type	day 60	I	1,911,025	Δ5,723 bp	Deletion	8635	ABC transporter substrate-binding protein	K01989
290	6	D60PM10	wild-type	day 60	I	791,679	A→T	K248* (AAA→TAA)	flrA	sigma-54 dependent transcriptional regulator	K10941
291	7	D300NM6	wild-type	day 300	I	129,760	(AAACAG)10→11	coding (217/1182 nt)	ftsY	signal recognition particle-docking protein FtsY	K03110
292	7	D300NM6	wild-type	day 300	I	394,383	G→A	S486L (TCG→TTG)	pepA	leucyl aminopeptidase	K01255
293	7	D300NM6	wild-type	day 300	I	835,793	G→A	R513H (CGC→CAC)	sucA	2-oxoglutarate dehydrogenase E1 component	K00164
294	7	D300NM6	wild-type	day 300	I	1,282,017	Δ29,643 bp	Deletion	23 genes		
295	7	D300NM6	wild-type	day 300	I	1,282,017	Δ29,643 bp	Deletion	5945	sodium-dependent transporter	K03308
296	7	D300NM6	wild-type	day 300	I	1,282,017	Δ29,643 bp	Deletion	5950	TIGR01621 family pseudouridine synthase	K06177
297	7	D300NM6	wild-type	day 300	I	1,282,017	Δ29,643 bp	Deletion	5955	CDP-alcohol phosphatidyltransferase family protein	-
298	7	D300NM6	wild-type	day 300	I	1,282,017	Δ29,643 bp	Deletion	5960	ATP-binding cassette domain-containing protein	K05779
299	7	D300NM6	wild-type	day 300	I	1,282,017	Δ29,643 bp	Deletion	5965	ABC transporter permease	K05778
300	7	D300NM6	wild-type	day 300	I	1,282,017	Δ29,643 bp	Deletion	5970	ABC transporter substrate-binding protein	K05777
301	7	D300NM6	wild-type	day 300	I	1,282,017	Δ29,643 bp	Deletion	hslJ	META domain-containing protein	K03668
302	7	D300NM6	wild-type	day 300	I	1,282,017	Δ29,643 bp	Deletion	5980	DUF1289 domain-containing protein	K06938
303	7	D300NM6	wild-type	day 300	I	1,282,017	Δ29,643 bp	Deletion	5985	hypothetical protein	-
304	7	D300NM6	wild-type	day 300	I	1,282,017	Δ29,643 bp	Deletion	5990	ABC transporter permease	K01992
305	7	D300NM6	wild-type	day 300	I	1,282,017	Δ29,643 bp	Deletion	5995	ABC transporter ATP-binding protein	K01990
306	7	D300NM6	wild-type	day 300	I	1,282,017	Δ29,643 bp	Deletion	6000	HlyD family efflux transporter periplasmic adaptor subunit	K01993
307	7	D300NM6	wild-type	day 300	I	1,282,017	Δ29,643 bp	Deletion	6005	serine transporter	K03837
308	7	D300NM6	wild-type	day 300	I	1,282,017	Δ29,643 bp	Deletion	6010	DMT family transporter	-
309	7	D300NM6	wild-type	day 300	I	1,282,017	Δ29,643 bp	Deletion	mgtE	magnesium transporter	K06213
310	7	D300NM6	wild-type	day 300	I	1,282,017	Δ29,643 bp	Deletion	evgS	transporter substrate-binding domain-containing protein	K07679
311	7	D300NM6	wild-type	day 300	I	1,282,017	Δ29,643 bp	Deletion	vieA	two-component system response regulator VieA	K13246

312	7	D300NM6	wild-type	day 300	I	1,282,017	Δ29,643 bp	Deletion	vieB	response regulator	-
313	7	D300NM6	wild-type	day 300	I	1,282,017	Δ29,643 bp	Deletion	6035	M9 family metallopeptidase	K01387
314	7	D300NM6	wild-type	day 300	I	1,282,017	Δ29,643 bp	Deletion	vesC	GlyGly-anchored extracellular serine protease VesC	-
315	7	D300NM6	wild-type	day 300	I	1,282,017	Δ29,643 bp	Deletion	6045	SgrR family transcriptional regulator	-
316	7	D300NM6	wild-type	day 300	I	1,282,017	Δ29,643 bp	Deletion	6050	hypothetical protein	-
317	7	D300NM6	wild-type	day 300	I	1,282,017	Δ29,643 bp	Deletion	6055	HAD family phosphatase	K07025
318	7	D300NM6	wild-type	day 300	I	1,462,862	C→T	G314D (GGT→GAT)	6745	hypothetical protein	-
319	7	D300NM6	wild-type	day 300	I	2,340,484	G→T	A82S (GCC→TCC)	btsR	two-component system response regulator BtsR	K02477
320	7	D300NM6	wild-type	day 300	I	2,777,147	Δ2 bp	coding (1404-1405/1824 nt)	12610	cyclic nucleotide-binding/CBS domain-containing protein	K07182
321	7	D300NM6	wild-type	day 300	I	2,779,903	G→A	G499D (GGT→GAT)	acs	acetate--CoA ligase	K01895
322	7	D300NM6	wild-type	day 300	II	94,972	(TCTGGT)18→17	coding (1014-1019/1248 nt)	14440	procyclic acidic repetitive family protein	-
323	7	D300NM6	wild-type	day 300	II	994,523	C→T	P259S (CCG→TCG)	focA	formate transporter FocA	K06212
324	7	D300NM6	wild-type	day 300	I	791,040	G→T	E35* (GAG→TAG)	flrA	sigma-54 dependent transcriptional regulator	K10941
325	7	D300NM6	wild-type	day 300	I	1,911,326	Δ5293 bp	Deletion	4 genes		
326	7	D300NM6	wild-type	day 300	I	1,911,326	Δ5293 bp	Deletion	8640	VF530 family DNA-binding protein	-
327	7	D300NM6	wild-type	day 300	I	1,911,326	Δ5293 bp	Deletion	8645	DUF412 domain-containing protein	K09899
328	7	D300NM6	wild-type	day 300	I	1,911,326	Δ5293 bp	Deletion	ackA	acetate kinase	K00925
329	7	D300NM6	wild-type	day 300	I	1,911,326	Δ5293 bp	Deletion	pta	phosphate acetyltransferase	K13788
330	7	D300NM6	wild-type	day 300	I	1,928,581	G→A	I268I (ATC→ATT)	8705	EAL domain-containing protein	-
331	7	D300NM6	wild-type	day 300	I	2,833,157	A→C	C202W (TGT→TGG)	12865	DegT/DnrJ/EryC1/StrS family aminotransferase	K13010
332	7	D300PM1	wild-type	day 300	I	791,040	G→T	E35* (GAG→TAG)	flrA	sigma-54 dependent transcriptional regulator	K10941
333	7	D300PM1	wild-type	day 300	I	1,911,326	Δ5293 bp	Deletion	4 genes		
334	7	D300PM1	wild-type	day 300	I	1,911,326	Δ5293 bp	Deletion	8640	VF530 family DNA-binding protein	-
335	7	D300PM1	wild-type	day 300	I	1,911,326	Δ5293 bp	Deletion	8645	DUF412 domain-containing protein	K09899
336	7	D300PM1	wild-type	day 300	I	1,911,326	Δ5293 bp	Deletion	ackA	acetate kinase	K00925
337	7	D300PM1	wild-type	day 300	I	1,911,326	Δ5293 bp	Deletion	pta	phosphate acetyltransferase	K13788
338	7	D300PM1	wild-type	day 300	I	1,928,581	G→A	I268I (ATC→ATT)	8705	EAL domain-containing protein	-
339	7	D300PM1	wild-type	day 300	I	2,833,157	A→C	C202W (TGT→TGG)	12865	DegT/DnrJ/EryC1/StrS family aminotransferase	K13010
340	7	D300PM1	wild-type	day 300	I	521	C→T	T174I (ACC→ATC)	dnaA	chromosomal replication initiator protein DnaA	K02313
341	7	D300PM1	wild-type	day 300	I	143,043	Δ1 bp	coding (53/459 nt)	690	RNA-binding protein	-
342	7	D300PM1	wild-type	day 300	I	207,993	(T)5→4	coding (434/630 nt)	995	response regulator transcription factor	-
343	7	D300PM1	wild-type	day 300	I	1,362,350	G→T	M12I (ATG→ATT)	citA	sensor histidine kinase	K02476
344	7	D300PM1	wild-type	day 300	I	2,961,962	Δ10 bp	coding (692-701/747 nt)	13490	amino acid ABC transporter substrate-binding protein	K16961
345	7	D300PM1	wild-type	day 300	II	993,225	G→T	A103E (GCG→GAG)	18305	LysR family transcriptional regulator	-
346	8	D300MT1	wild-type	day 300	II	963,770	(AGGGAGCAGATTGGGG)1→2	intergenic (+250/+335)	-	-	-
347	8	D300MT1	wild-type	day 300	I	460,748	C→A	G311C (GGT→TGT)	2240	inorganic phosphate transporter	K03306
348	8	D300MT1	wild-type	day 300	I	573,120	C→T	S143F (TCT→TTT)	2680	NupC/NupG family nucleoside CNT transporter	K03317
349	8	D300MT1	wild-type	day 300	I	806,455	G→A	W15* (TGG→TAG)	fliQ	flagellar biosynthesis protein FliQ	K02420
350	8	D300MT1	wild-type	day 300	I	1,025,407	T→A	L207H (CTT→CAT)	cysB	HTH-type transcriptional regulator CysB	K13634
351	8	D300MT1	wild-type	day 300	I	1,025,639	G→A	M284I (ATG→ATA)	cysB	HTH-type transcriptional regulator CysB	K13634
352	8	D300MT1	wild-type	day 300	I	1,090,287	A→G	D111G (GAC→GGC)	ompT	porin OmpT	K10940
353	8	D300MT1	wild-type	day 300	I	1,914,057	G→A	G331D (GGT→GAT)	ackA	acetate kinase	K00925
354	8	D300MT1	wild-type	day 300	I	1,923,642	A→T	intergenic (-301/-323)	-	-	-
355	8	D300MT1	wild-type	day 300	I	2,836,261	Δ5 bp	coding (24-28/1392 nt)	12875	phosphomannomutase CpsG	K01840
356	8	D300MT1	wild-type	day 300	II	698,941	Δ18 bp	coding (689-706/795 nt)	phhA	phenylalanine 4-monooxygenase	K00500

357	8	D300NM7	wild-type	day 300	I	460,748	C→A	G311C (GGT→TGT)	2240	inorganic phosphate transporter	K03306
358	8	D300NM7	wild-type	day 300	I	573,120	C→T	S143F (TCT→TTT)	2680	NupC/NupG family nucleoside CNT transporter	K03317
359	8	D300NM7	wild-type	day 300	I	806,455	G→A	W15* (TGG→TAG)	fliQ	flagellar biosynthesis protein FliQ	K02420
360	8	D300NM7	wild-type	day 300	I	1,025,407	T→A	L207H (CTT→CAT)	cysB	HTH-type transcriptional regulator CysB	K13634
361	8	D300NM7	wild-type	day 300	I	1,025,639	G→A	M284I (ATG→ATA)	cysB	HTH-type transcriptional regulator CysB	K13634
362	8	D300NM7	wild-type	day 300	I	1,090,287	A→G	D111G (GAC→GGC)	ompT	porin OmpT	K10940
363	8	D300NM7	wild-type	day 300	I	1,914,057	G→A	G331D (GGT→GAT)	ackA	acetate kinase	K00925
364	8	D300NM7	wild-type	day 300	I	1,923,642	A→T	intergenic (-301/-323)	-	-	-
365	8	D300NM7	wild-type	day 300	I	2,836,261	Δ5 bp	coding (24-28/1392 nt)	12875	phosphomannomutase CpsG	K01840
366	8	D300NM7	wild-type	day 300	II	698,941	Δ18 bp	coding (689-706/795 nt)	phhA	phenylalanine 4-monooxygenase	K00500
367	8	D300PM2	wild-type	day 300	I	460,748	C→A	G311C (GGT→TGT)	2240	inorganic phosphate transporter	K03306
368	8	D300PM2	wild-type	day 300	I	573,120	C→T	S143F (TCT→TTT)	2680	NupC/NupG family nucleoside CNT transporter	K03317
369	8	D300PM2	wild-type	day 300	I	806,455	G→A	W15* (TGG→TAG)	fliQ	flagellar biosynthesis protein FliQ	K02420
370	8	D300PM2	wild-type	day 300	I	1,025,407	T→A	L207H (CTT→CAT)	cysB	HTH-type transcriptional regulator CysB	K13634
371	8	D300PM2	wild-type	day 300	I	1,025,639	G→A	M284I (ATG→ATA)	cysB	HTH-type transcriptional regulator CysB	K13634
372	8	D300PM2	wild-type	day 300	I	1,090,287	A→G	D111G (GAC→GGC)	ompT	porin OmpT	K10940
373	8	D300PM2	wild-type	day 300	I	1,914,057	G→A	G331D (GGT→GAT)	ackA	acetate kinase	K00925
374	8	D300PM2	wild-type	day 300	I	1,923,642	A→T	intergenic (-301/-323)	-	-	-
375	8	D300PM2	wild-type	day 300	I	2,836,261	Δ5 bp	coding (24-28/1392 nt)	12875	phosphomannomutase CpsG	K01840
376	8	D300PM2	wild-type	day 300	II	698,941	Δ18 bp	coding (689-706/795 nt)	phhA	phenylalanine 4-monooxygenase	K00500
377	9	VC50	ΔmutS	day 60	I	2,513,434	Δ2,586 bp	coding (4-2589/2589 nt)	mutS	DNA mismatch repair protein MutS	
378	9	VC50	ΔmutS	day 60	I	83,950	A→G	K133K (AAA→AAG)	plsB	glycerol-3-phosphate 1-O-acyltransferase PlsB	
379	9	VC50	ΔmutS	day 60	I	792,321	(G)$_{7\rightarrow 8}$	coding (1384/1467 nt)	flrA	sigma-54 dependent transcriptional regulator	
380	9	VC50	ΔmutS	day 60	I	907,063	G→A	V211V (GTG→GTA)	4280	ketoacyl-ACP synthase III	
381	9	VC50	ΔmutS	day 60	I	952,820	(T)$_{7\rightarrow 6}$	coding (280/585 nt)	yfbR	5'-deoxynucleotidase	
382	9	VC50	ΔmutS	day 60	I	1,339,407	C→T	L232F (CTC→TTC)	nspC	carboxynorspermidine decarboxylase	
383	9	VC50	ΔmutS	day 60	I	1,462,493	C→T	R437H (CGT→CAT)	6745	hypothetical protein	
384	9	VC50	ΔmutS	day 60	I	1,684,849	T→C	E28G (GAA→GGA)	7625	SLC13 family permease	
385	9	VC50	ΔmutS	day 60	I	1,858,537	(T)$_{7\rightarrow 8}$	coding (206/906 nt)	8390	lysine exporter LysO family protein	
386	9	VC50	ΔmutS	day 60	I	1,906,979	Δ8,347 bp	Deletion	8 genes		
387	9	VC50	ΔmutS	day 60	I	1,906,979	Δ8,347 bp	Deletion	8620	diguanylate cyclase	
388	9	VC50	ΔmutS	day 60	I	1,906,979	Δ8,347 bp	Deletion	8625	ABC transporter ATP-binding protein	
389	9	VC50	ΔmutS	day 60	I	1,906,979	Δ8,347 bp	Deletion	8630	ABC transporter permease	
390	9	VC50	ΔmutS	day 60	I	1,906,979	Δ8,347 bp	Deletion	8635	ABC transporter substrate-binding protein	
391	9	VC50	ΔmutS	day 60	I	1,906,979	Δ8,347 bp	Deletion	8640	VF530 family DNA-binding protein	
392	9	VC50	ΔmutS	day 60	I	1,906,979	Δ8,347 bp	Deletion	8645	DUF412 domain-containing protein	
393	9	VC50	ΔmutS	day 60	I	1,906,979	Δ8,347 bp	Deletion	ackA	acetate kinase	
394	9	VC50	ΔmutS	day 60	I	1,906,979	Δ8,347 bp	Deletion	pta	phosphate acetyltransferase	
395	9	VC50	ΔmutS	day 60	I	2,207,314	C→T	pseudogene (402/681 nt)	9965	IS5 family transposase	
396	9	VC50	ΔmutS	day 60	I	2,388,037	G→A	A104V (GCT→GTT)	10750	MarR family transcriptional regulator	
397	9	VC50	ΔmutS	day 60	I	2,442,072	T→C	M283V (ATG→GTG)	10955	Fe(3+) ABC transporter substrate-binding protein	
398	9	VC50	ΔmutS	day 60	I	2,469,054	G→A	W127* (TGG→TGA)	11060	TetR/AcrR family transcriptional regulator	
399	9	VC50	ΔmutS	day 60	I	2,810,758	(T)$_{9\rightarrow 8}$	coding (212/1200 nt)	manA	mannose-6-phosphate isomerase, class I	
400	9	VC50	ΔmutS	day 60	I	2,960,688	C→T	L161L (CTG→CTA)	13480	amino acid ABC transporter ATP-binding protein	
401	9	VC50	ΔmutS	day 60	II	467,994	(C)$_{11\rightarrow 10}$	intergenic (+20/-162)	-	-	

402	9	VC50	ΔmutS	day 60	II	477,002	$(G)_{9\to8}$	coding (119/942 nt)	16010	M23 family metallopeptidase
403	9	VC50	ΔmutS	day 60	II	698,427	C→T	P59S (CCA→TCA)	phhA	phenylalanine 4-monooxygenase
404	9	VC50	ΔmutS	day 60	II	973,965	$(A)_{7\to6}$	coding (1077/1239 nt)	18225	GGDEF domain-containing protein
405	9	VC50	ΔmutS	day 60	II	1,043,835	G→A	intergenic (-37/+99)	-	-
406	9	VC50	ΔmutS	day 60	II	1,043,850	C→T	intergenic (-52/+84)	-	-
407	9	VC50	ΔmutS	day 60	II	1,043,859	G→A	intergenic (-61/+75)	-	-
408	9	VC50	ΔmutS	day 60	II	1,043,869	T→C	intergenic (-71/+65)	-	-
409	9	VC50	ΔmutS	day 60	II	1,043,871	C→T	intergenic (-73/+63)	-	-
410	9	VC50	ΔmutS	day 60	II	1,043,887	T→A	intergenic (-89/+47)	-	-
411	10	VC51	ΔmutS	day 60	I	2,513,434	Δ2,586 bp	coding (4-2589/2589 nt)	mutS	DNA mismatch repair protein MutS
412	10	VC51	ΔmutS	day 60	I	316	$(CTGCGC)_{1\to2}$	coding (316/1404 nt)	dnaA	chromosomal replication initiator protein DnaA
413	10	VC51	ΔmutS	day 60	I	473,435	$(A)_{7\to8}$	coding (243/813 nt)	cpdA	3',5'-cyclic-AMP phosphodiesterase
414	10	VC51	ΔmutS	day 60	I	549,405	A→G	I628I (ATT→ATC)	gltB	glutamate synthase large subunit
415	10	VC51	ΔmutS	day 60	I	771,085	A→G	F118L (TTC→CTC)	3620	M48 family metallopeptidase
416	10	VC51	ΔmutS	day 60	I	792,321	$(G)_{7\to8}$	coding (1384/1467 nt)	flrA	sigma-54 dependent transcriptional regulator
417	10	VC51	ΔmutS	day 60	I	1,339,407	C→T	L232F (CTC→TTC)	nspC	carboxynorspermidine decarboxylase
418	10	VC51	ΔmutS	day 60	I	1,345,295	$(G)_{6\to7}$	coding (2939/6756 nt)	6175	cadherin domain-containing protein
419	10	VC51	ΔmutS	day 60	I	1,417,112	G→A	P180L (CCG→CTG)	6515	sn-glycerol-3-phosphate import ATP-binding protein UgpC
420	10	VC51	ΔmutS	day 60	I	1,878,122	G→A	A137V (GCC→GTC)	hisC	histidinol-phosphate transaminase
421	10	VC51	ΔmutS	day 60	I	1,913,426	C→T	H121Y (CAC→TAC)	ackA	acetate kinase
422	10	VC51	ΔmutS	day 60	I	1,921,803	T→A	R513S (AGA→AGT)	8685	peptide ABC transporter substrate-binding protein
423	10	VC51	ΔmutS	day 60	I	1,956,804	T→C	E8E (GAA→GAG)	recR	recombination mediator RecR
424	10	VC51	ΔmutS	day 60	I	2,207,314	C→T	pseudogene (402/681 nt)	9965	IS5 family transposase
425	10	VC51	ΔmutS	day 60	I	2,499,005	T→C	E65E (GAA→GAG)	oadA	sodium-extruding oxaloacetate decarboxylase subunit alpha
426	10	VC51	ΔmutS	day 60	I	2,559,306	T→C	intergenic (+173/-84)	-	-
427	10	VC51	ΔmutS	day 60	I	2,869,187	G→A	G193G (GGC→GGT)	murP	PTS N-acetylmuramic acid transporter subunit IIBC
428	10	VC51	ΔmutS	day 60	II	179,134	T→C	intergenic (+44/+24)	-	-
429	10	VC51	ΔmutS	day 60	II	467,994	$(C)_{11\to10}$	intergenic (+20/-162)	-	-
430	10	VC51	ΔmutS	day 60	II	582,508	A→G	V19A (GTC→GCC)	16495	DMT family transporter
431	10	VC51	ΔmutS	day 60	II	584,989	$(G)_{7\to8}$	intergenic (-124/+530)	-	-
432	10	VC51	ΔmutS	day 60	II	698,791	$(G)_{5\to6}$	coding (539/795 nt)	phhA	phenylalanine 4-monooxygenase
433	10	VC51	ΔmutS	day 60	II	765,644	T→C	F123L (TTC→CTC)	viaA	ATPase RavA stimulator ViaA
434	10	VC51	ΔmutS	day 60	II	958,385	G→A	V771V (GTC→GTT)	18155	bifunctional acetate--CoA ligase family protein
