# Supplementary material for: Flagella-related gene mutations in Vibrio cholerae during extended cultivation in nutrient-limited media impair cell motility and prolong culturability
Source: mSystems. 2023 Aug 29;8(5):e00109-23. doi: 10.1128/msystems.00109-23 (PMC10654082; doi:10.1128/msystems.00109-23)
Supplement: Supplemental Text — Supplemental methods. [file msystems.00109-23-s0010.doc]

Supplementary Text

Measurement of reversion frequency to motility

The frequencies of reversion from partially motile or non-motile to motile phenotypes were determined using the pour plate technique (49). Briefly, an overnight culture of the partially motile or non-motile isolate in LB was diluted and added to motility agar; this was autoclaved and cooled to 42°C to achieve a final density of 200–400 colonies per plate, allowed to solidify at 20 – 30°C for 2 h, and incubated at 37°C for 24 h.

Phenotypic tests to select a variety of variants

Prolonged cultures in M9 were sampled at the indicated time points and plated on LB agar plates at 37°C overnight. Colonies were picked and examined for phenotypic assays.

1. Motility assay

See Material and Methods in main text.

1. Hemolysis assay

The individual colonies were stabbed on blood agar plates (LB containing 1.5% agar and 5% sheep blood) and incubated at 37°C for 24 h. Diameters of the hemolysis zones were measured.

1. Proteolytic activity assay

The presence of proteolytic activity was examined as described previously (50). Briefly, the individual colonies were stabbed on LB agar containing 3% skim milk and incubated at 37°C for 24 h. Diameters of clearing zones were measured.

1. Catalase activity assay

Catalase activity was measured as previously described (51). Briefly, 1.4 ml overnight culture in LB was centrifuged, and the pellet was weighed and resuspended in 100 μl PBS. The suspension was placed in a 13 mm × 100 mm tube; 100 μl of 1% Triton X-100 was added, followed by the addition of 100 μl of 30 % H_2_O_2_ solution, thorough mixing, and incubation at room temperature for 15 min. The height of bubble formation was measured in millimeters. Catalase activity (units/mg) was determined using calibration curves constructed with standard catalase solutions.

1. Biofilm formation assay

The biofilm formation assay was performed as reported previously with some modifications (52). Briefly, 20 μl of the overnight culture was added to 2 ml LB in a 15 ml polystyrene tube. The tubes were incubated at 37°C for 72 h under static conditions. The tubes were washed three times with distilled water to remove non-adherent cells. Biofilms were stained by the addition of 2 ml of 1% crystal violet (Wako) for 30 min, washed three times with distilled water, restained with crystal violet, solubilized with 2 ml of dimethyl sulfoxide (DMSO; VWR Chemicals), and quantified by measuring the OD_595_ using a microplate reader (Multiskan FC, Thermo, Waltham, MA, USA).

Measurement of cholera toxin (CT) production

Cultures grown overnight in LB (50 μl) were added to 5 ml AKI broth containing 15 g/l peptone, 4 g/l yeast extract, 5 g/l NaCl, and 3 g NaHCO_3_. The tubes were incubated for 20 h at 37°C under static conditions. The culture supernatant was recovered by centrifugation for 5 min. The concentration of CT in the culture supernatant was quantified using the GM1-ganglioside ELISA method (53).

**REFERENCES**

49. Mostow P, Richardson K. High-frequency spontaneous mutation of classical *Vibrio cholerae* to a nonmotile phenotype. Infect Immun*.* 1990; 58:3633–3639.

50. Hulsmann A, Rosche TM, Kong IS, Hassan HM, Beam DM, Oliver JD. RpoS-dependent stress response and exoenzyme production in *Vibrio vulnificus.* Appl Environ Microbiol*.* 2003; 69:6114–6120.

51. Iwase T, Tajima A, Sugimoto S, Okuda KI, Hironaka I, Kamata Y *et al*. A simple assay for measuring catalase activity: a visual approach. Sci Rep*.* 2013; 3:3081.

1. Bomchil N, Watnick P, Kolter R. Identification and characterization of a *Vibrio cholerae* gene, mbaA, involved in maintenance of biofilm architecture. J Bacteriol*.* 2003; 185:1384–1390.
2. Centers for Disease Control and Prevention. Laboratory Methods for the Diagnosis of *Vibrio cholerae*—VI. Laboratory identification of *Vibrio cholerae*. <https://www.cdc.gov/cholera/pdf/Laboratory-Methods-for-the-Diagnosis-of-Vibrio-cholerae-chapter-6.pdf. Cited 17 May 2022>.
